# Supplementary material for: DNA origami scaffold for studying intrinsically disordered proteins of the nuclear pore complex
Source: Nat Commun. 2018 Mar 2;9:902. doi: 10.1038/s41467-018-03313-w (PMC5834454; doi:10.1038/s41467-018-03313-w)
Supplement: Supplementary file 1 — Supplementary Information [file 41467_2018_3313_MOESM1_ESM.pdf]

SUPPLEMENTARY INFORMATION

DNA origami scaffold for studying intrinsically disordered proteins of the nuclear pore complex

Philip Ketterer[1,*], Adithya N. Ananth[2,*], Diederik S. Laman Trip[2], Ankur Mishra[3], Eva Bertosin[1], Mahipal Ganji[2], Jaco van der Torre[2], Patrick Onck[3], Hendrik Dietz[1‡], Cees Dekker[2‡]

[1]Physik Department and Institute for Advanced Study, Technische Universität München, Am Coulombwall 4a, Garching bei München, D-85748, Germany
[2]Department of Bionanoscience, Kavli Institute of Nanoscience, Delft University of Technology, Van der Maasweg 9, 2629 HZ Delft, The Netherlands.
[3]Zernike Institute for Advanced Materials, University of Groningen, Nijenborgh 4, 9747AG, Groningen, The Netherlands

[‡]corresponding author's e-mail addresses: c.dekker@tudelft.nl, dietz@tum.de
*These authors contributed equally to this work.

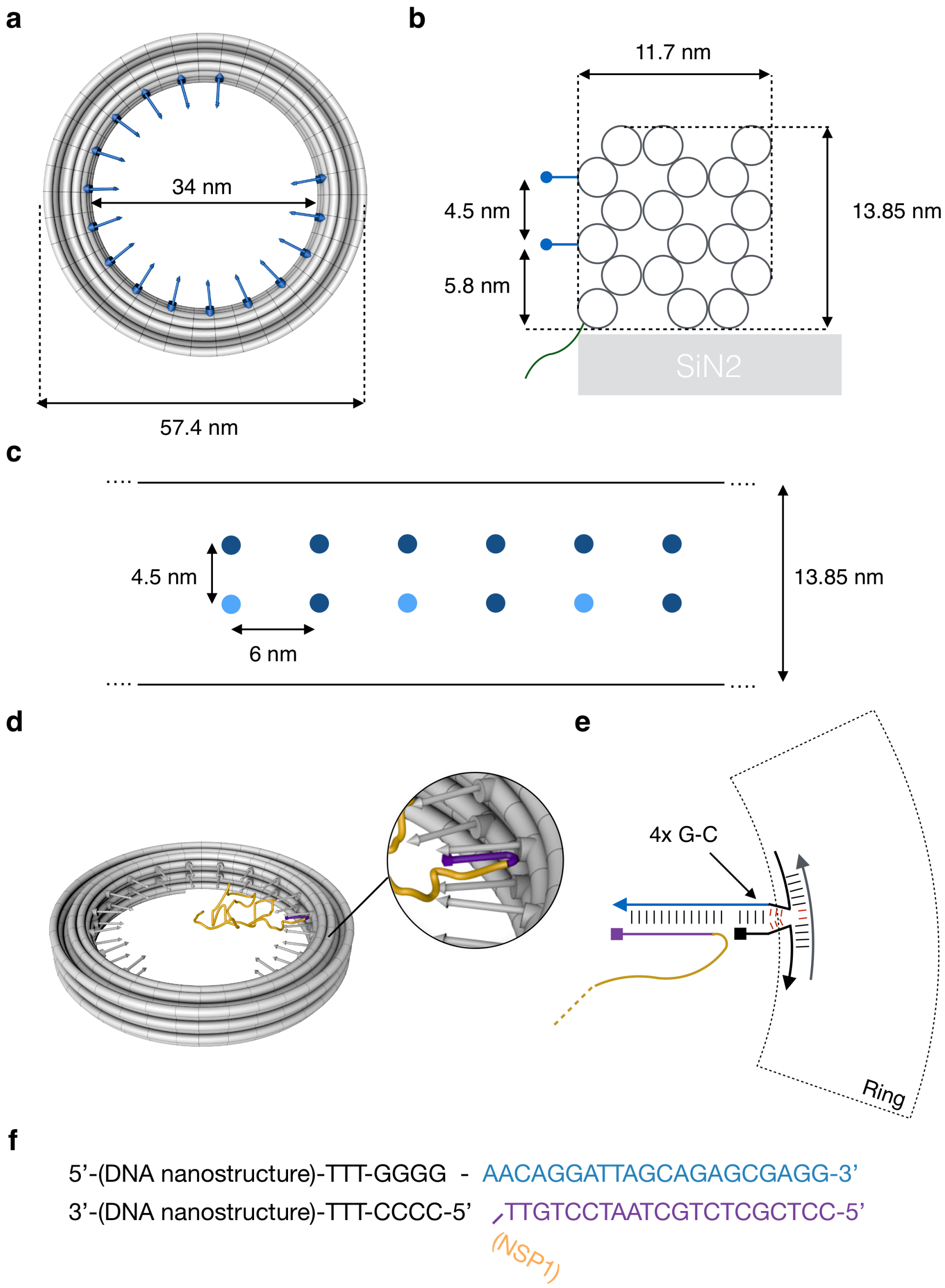

Supplementary Figure 1. Geometry of DNA origami ring and protein attachment. a, Schematic representation of DNA origami ring with indicated dimensions. Attachment anchors depicted in blue. **b,** Schematic cross section of the ring. Attachment anchors are depicted in blue and the starting position of the leash in green. **c,** Schematic view onto a part of the inner surface of the ring indicating the geometry of the attachment anchors. Light blue circles indicate anchors used for the 8 attachment sites variant, dark blue and light blue circles indicate positions of all 32 attachment anchors. **d,** Schematic representation of DNA ring with one protein bound. DNA oligonucleotide attached to the protein is depicted in purple. **e,** Schematic sketch of the design of one attachment anchor. Red lines indicate unpaired nucleotides. **f,** Attachment anchor sequences.

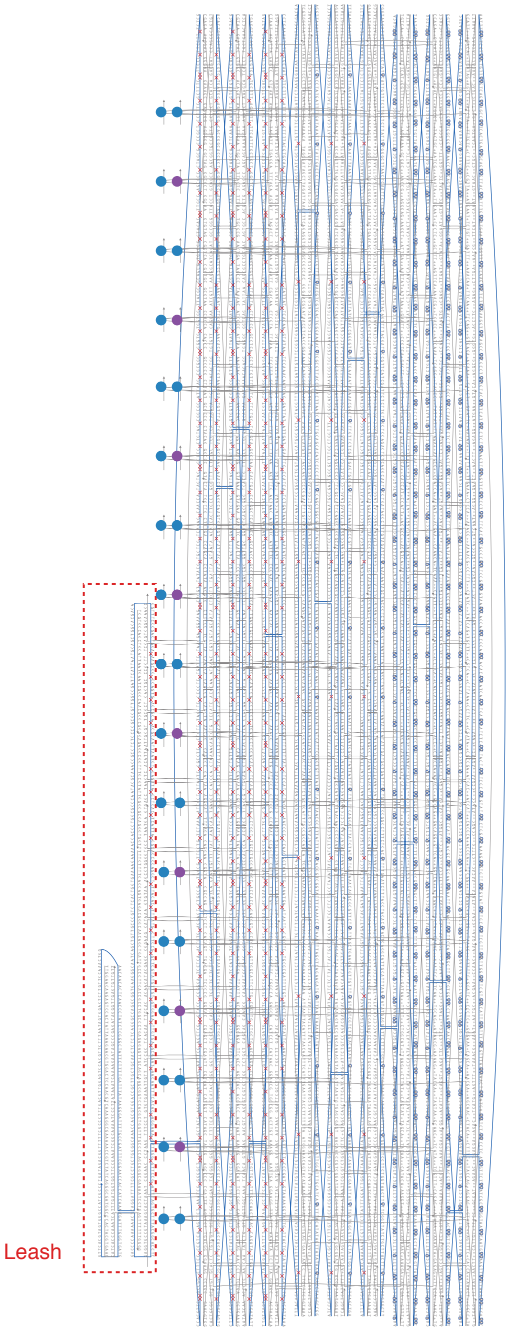

Supplementary Figure 2. Scaffold / staple layout of the DNA ring. Generated with caDNAno v0.2. Purple dots indicate positions of attachment anchors of the variant with 8 anchors, blue and purple dots indicate positions of all 32 attachment anchors.

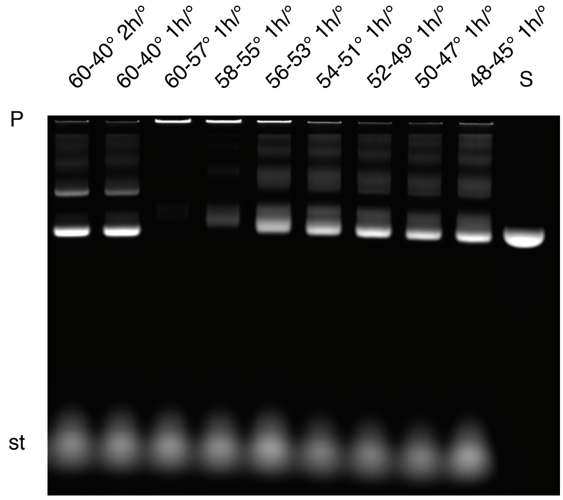

Supplementary Figure 3. Electrophoretic mobility analysis of the ring. Laser-scanned image of a 2% agarose gel with 11mM $MgCl_2$ run on an iced water-bath. S=scaffold DNA, folding ramps as indicated. ‘P’ denotes the pockets and ‘st’ the excess staple oligonucleotides. Image was globally auto-levelled below the pockets.

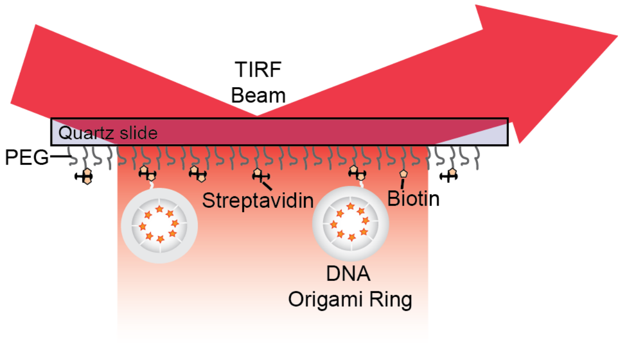

Supplementary Figure 4. Schematic diagram showing TIRF imaging of Cy5-labelled DNA origami rings. The rings are attached to the Quartz surface by biotin-streptavidin-biotin interactions.

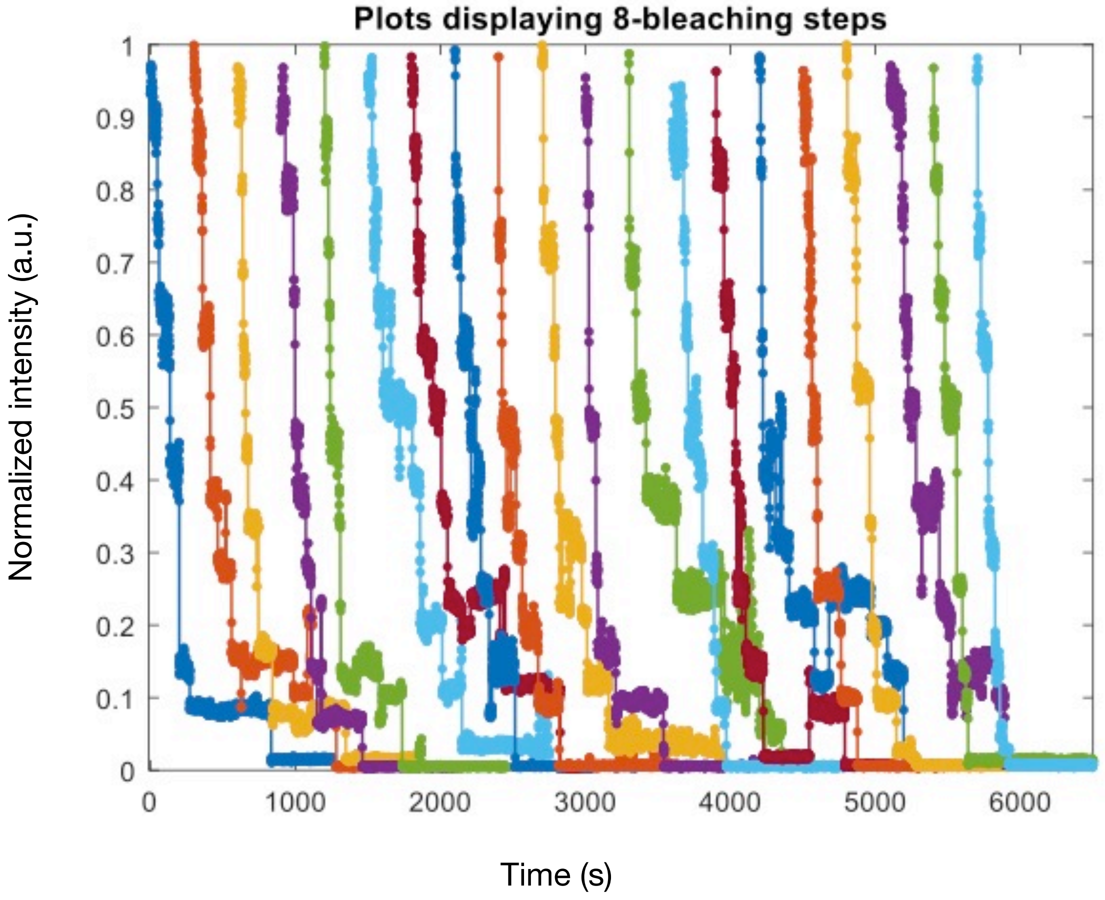

Supplementary Figure 5. Exemplary TIRF bleaching curves showing 8 bleaching steps. Every plot is shifted in time by 300s for visualization. Intensity is normalized to the maximum intensity.

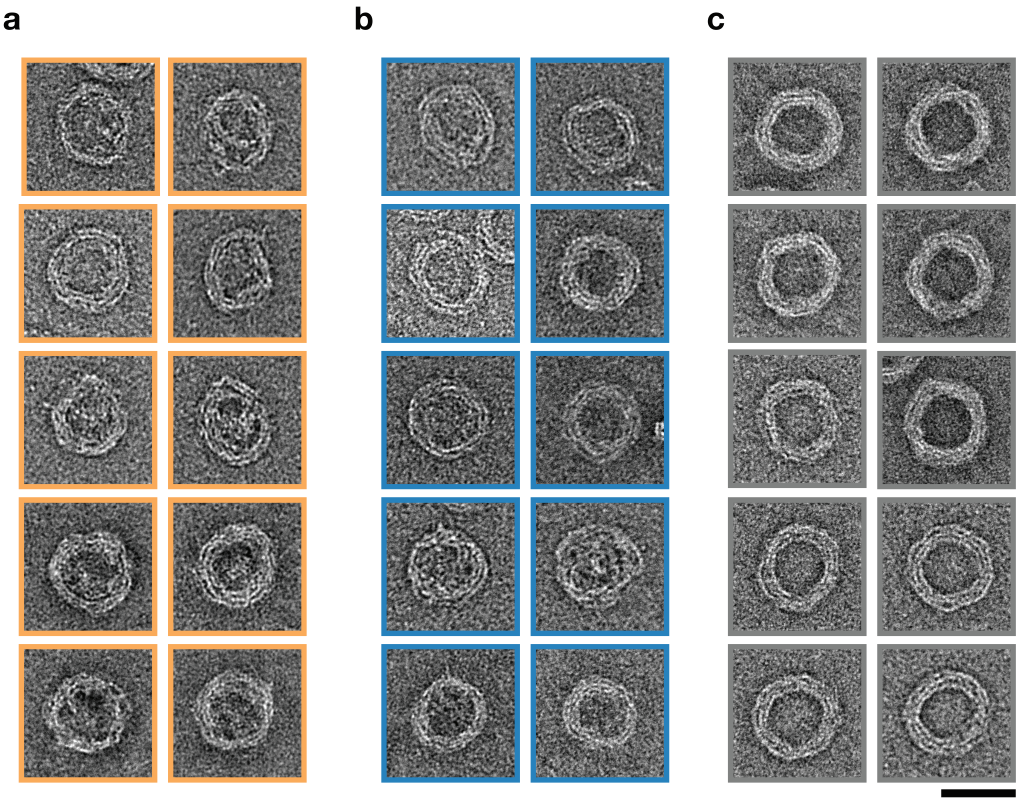

Supplementary Figure 6. Exemplary negative-stain TEM particles. Samples: DNA ring with 32-NSP1 (**a**), with 32-NSP1-S (**b**), and with no protein (**c**)**.** Scale bar = 50 nm.

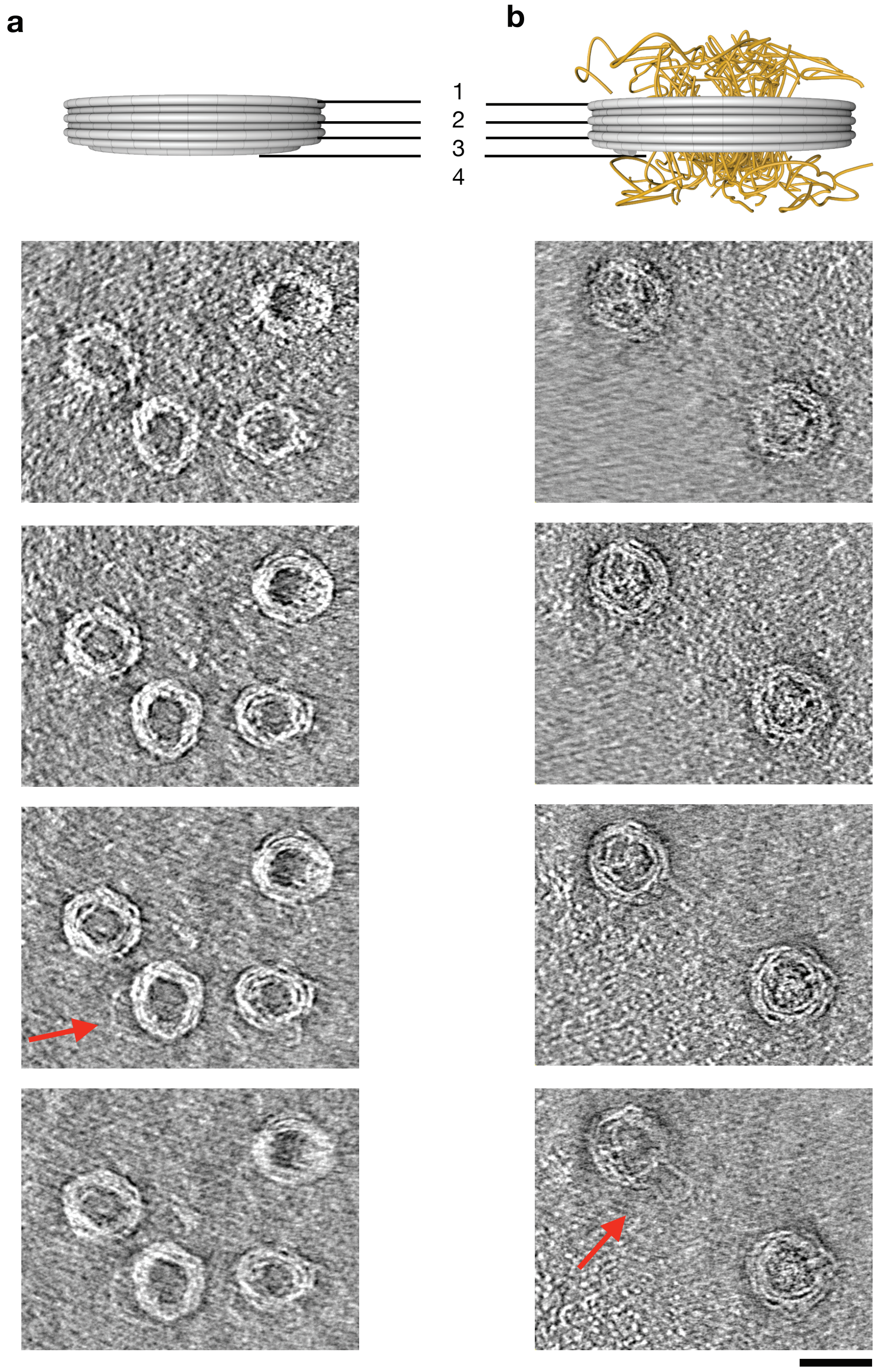

Supplementary Figure 7. Tomography of bare ring without proteins and of 32-NSP-1. Schematic representation of DNA ring without (**a**) and with NSP1 (**b**). Numbers indicate positions of slices of reconstruction from tomography data shown below top images at position 1; bottom images at position 4). Red arrows indicate the bottom leash of DNA rings. Scale bar = 50 nm.

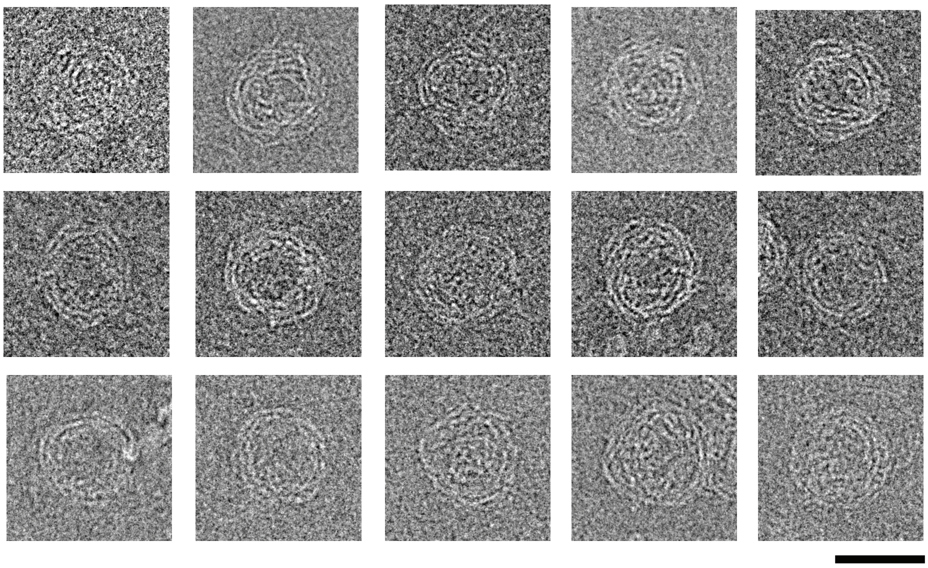

Supplementary Figure 8. Exemplary cryo-EM single particles of the sample 32-NSP1. Scale bar = 50 nm.

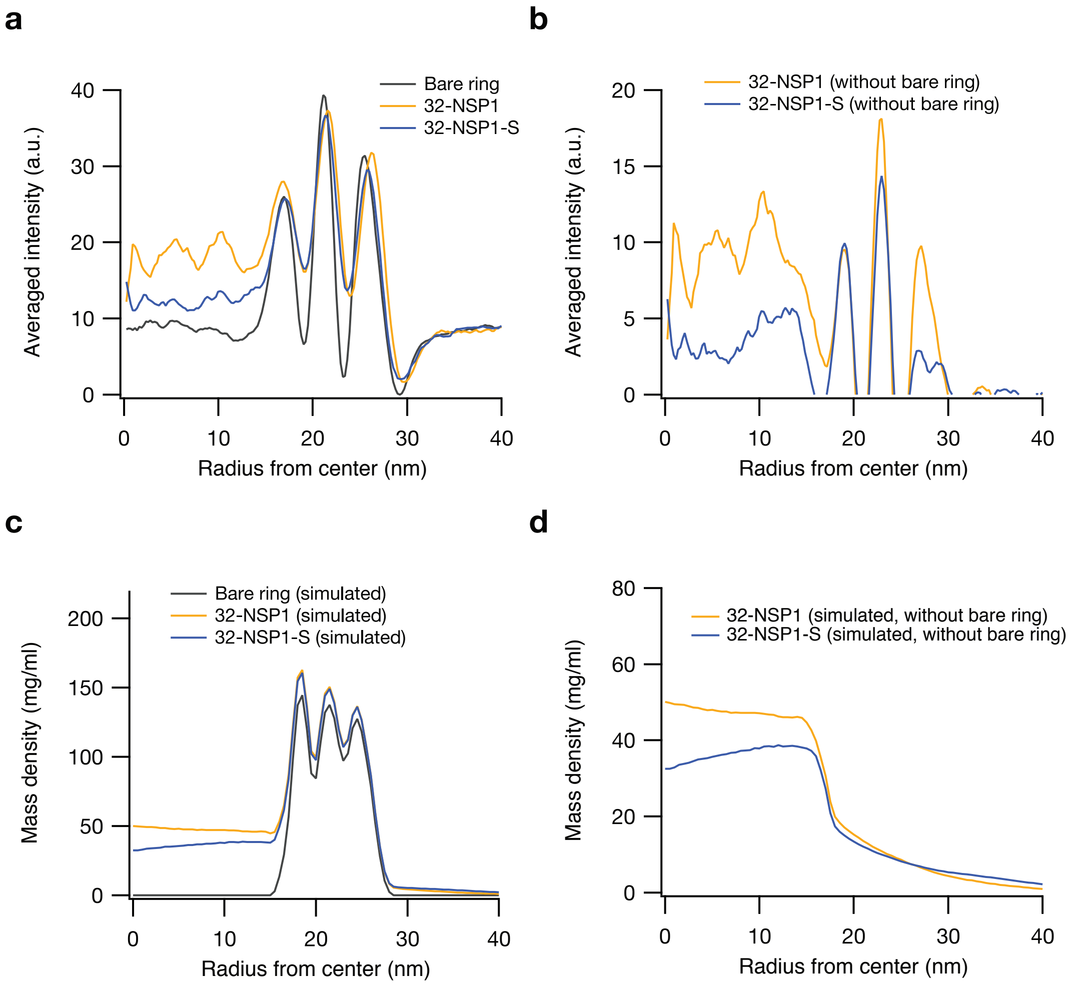

Supplementary Figure 9. Comparison of densities from cryo-EM and MD simulations. a, Circularly averaged radial intensity profiles obtained from the cryo-EM average micrographs shown in Fig. 3b containing the ring as well as the proteins. **b,** As in a, but the profile of the bare ring was subtracted from the profiles with proteins. Note that residual density oscillations for radii 17-28 nm are observed as these are difference signals from strongly oscillating intensities (see **a**) that are slightly misaligned. **c**, Time-averaged radial density distribution of the ring-nanopore system with NSP1 and NSP1-S, averaged in the circumferential direction and axial direction for $|z| < 25$ nm. The bare ring density was calculated from a pdb file obtained from a CanDo simulation. **d**, As in **c**, but only the protein density is shown. The data shows a higher mass density for rings with NSP1 compared to rings with NSP1-S for the same number (32) of FG-Nup copies.

a b

c

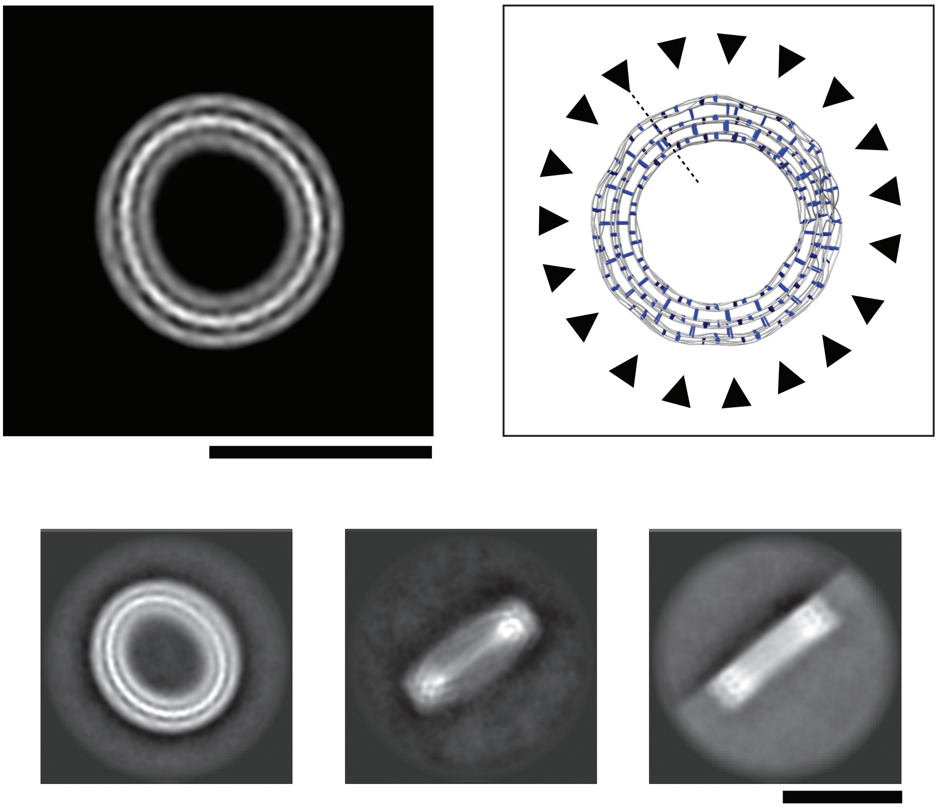

Supplementary Figure 10. Comparison of cryo-EM 2D class averages with predicted structure. a, Rotationally aligned reference-free class average of DNA ring with 32 attachment sites without attached protein calculated with Relion2[3]. Number of averaged particles is 2757. White arrows indicate density spikes. Scale bar = 50 nm. **b**, CanDo simulation of DNA ring. Crossovers are depicted in blue; arrows indicate their positions. **c**, Other class averages obtained from the same measurement as in **a.** Numbers of averaged particles are (f.l.t.r.) 2670, 1228, and 551. Scale bar = 50 nm.

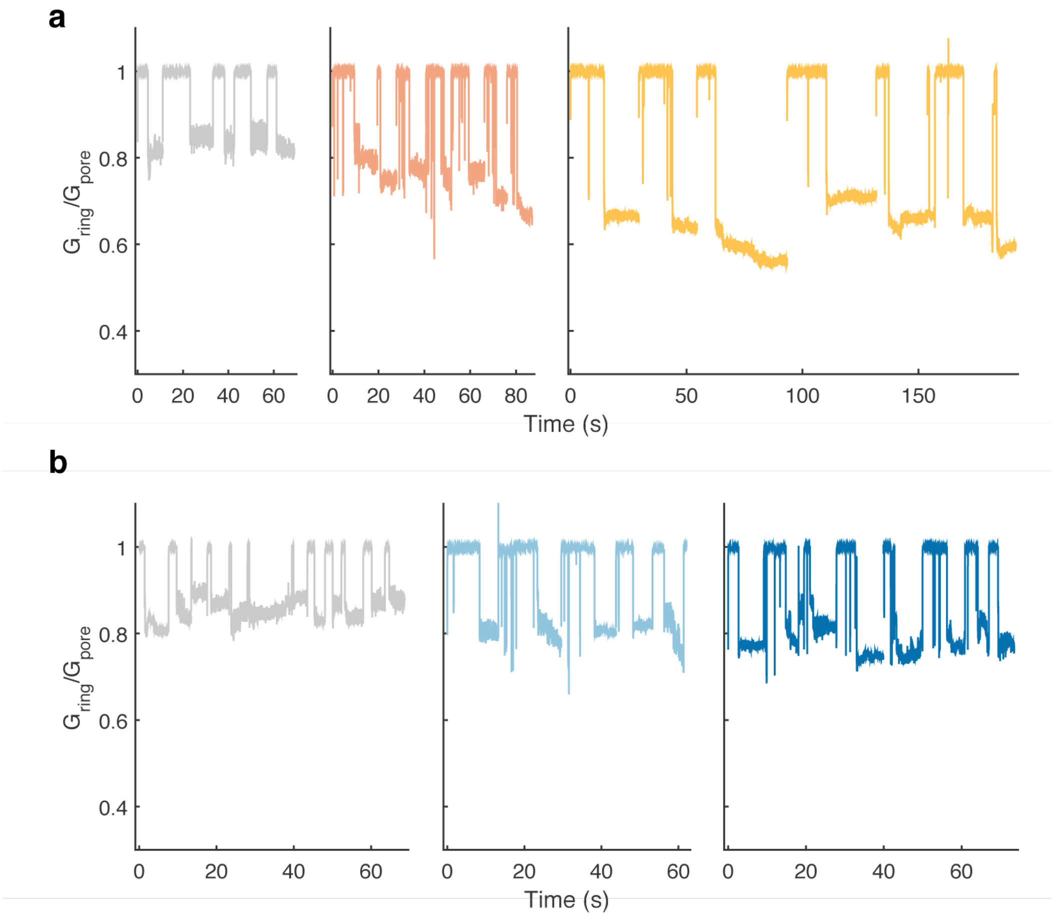

Supplementary Figure 11. Exemplary ionic current traces for ring-docking events. Reduced conductance (G_{ring}/G_{pore}) traces over time for DNA rings hat are docking onto a nanopore (see SI methods). **a,** Data for bare ring (gray), 8-NSP1 (red), 32-NSP1 (yellow). **b,** bare ring (gray), 8-NSP1-S (lightblue), 32-NSP1-S (blue).

a

b

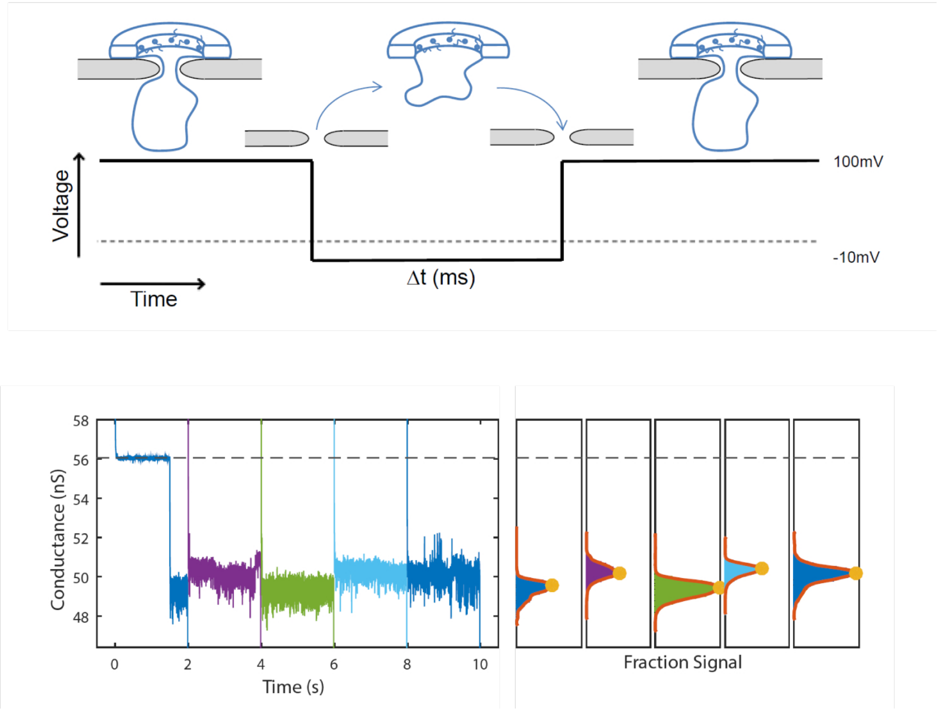

Supplementary Figure 12. Ionic current traces of multiple re-docking of a DNA ring on a nanopore. a, Schematic representation showing the DNA ring re-docking on the nanopore by temporarily switching the applied voltage from 100 mV to -10 mV for Δt = 10 ms (every 2 s). **b,** Left: Filtered (1kHz low-pass Gaussian) continuous ionic current trace showing the conductance of the DNA ring, displayed in different colors after each re-docking. Right: Smoothened trace histogram (red) and fitted histogram peaks (yellow) for the data plotted on the left. Gray dotted line indicates the average nanopore conductance at the moment just before the ring is first docked.

a

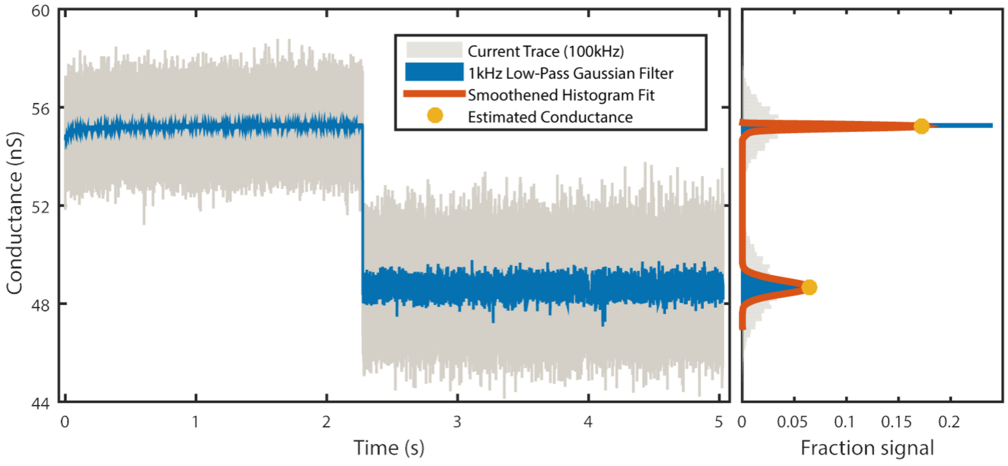

b

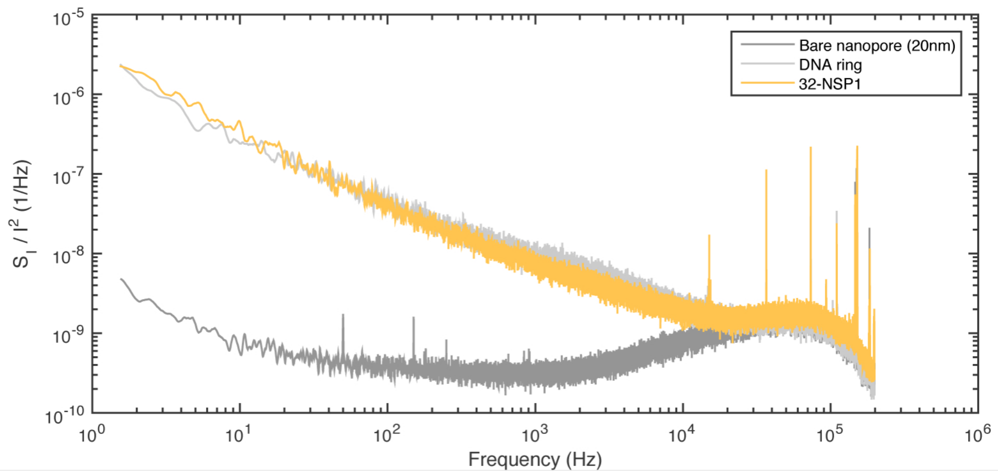

Supplementary Figure 13. Noise comparison of the bare nanopore and DNA ring docked on a nanopore. a, Raw ionic current trace of the bare nanopore and of a docked DNA ring, showing the filtered (1 kHz low-pass Gaussian, blue) and full bandwidth (100 kHz, gray) data, together with a smoothened trace histogram and fitted histogram peaks (yellow). The bare nanopore conductance is 55.23 nS and the docked DNA ring conductance is 48.65 nS. **b,** Normalized power spectral density of the measured ionic current trace for a bare nanopore (dark gray), a nanopore with docked bare ring (light gray), and a docked 32-NSP1 ring (yellow). Typical translocation events are observed near 1 kHz. The noise spikes at frequencies above 20 kHz are inherent to the system setup and shared among all three traces.

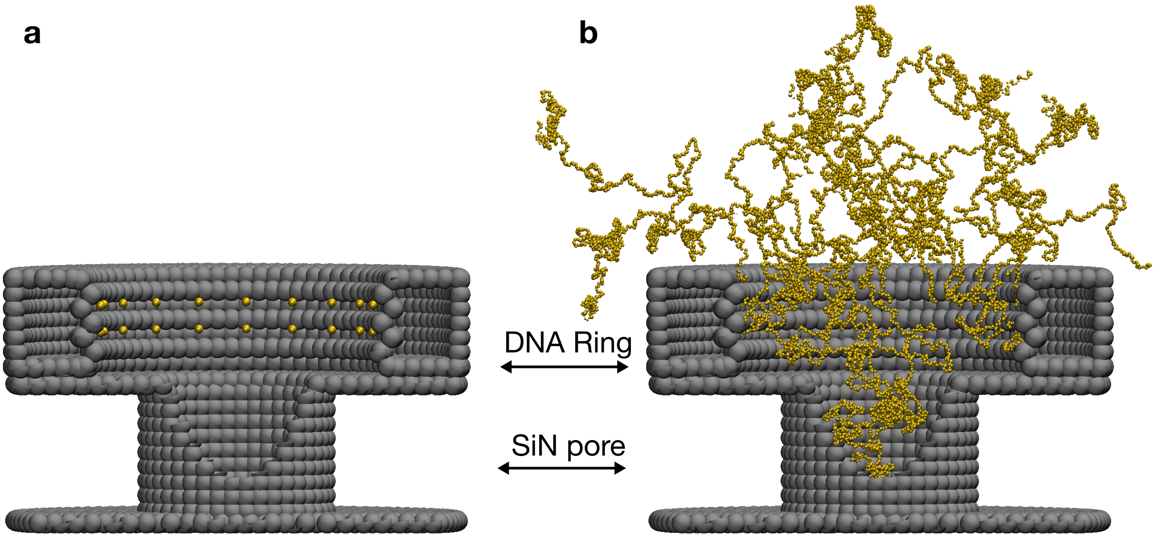

Supplementary Figure 14. MD simulations of DNA ring with attached Nups on a solid-state nanopore. a, Cut-through of the DNA ring model with a height of 13.85 nm and a diameter of 36 nm, placed on top of a solid-state nanopore with a height and diameter of 20 nm. The positions of the attachment anchors for the Nups are indicated in yellow. **b**, Cut-through of a snapshot of a MD simulation with in total 32 NSP1 proteins tethered inside the DNA ring.

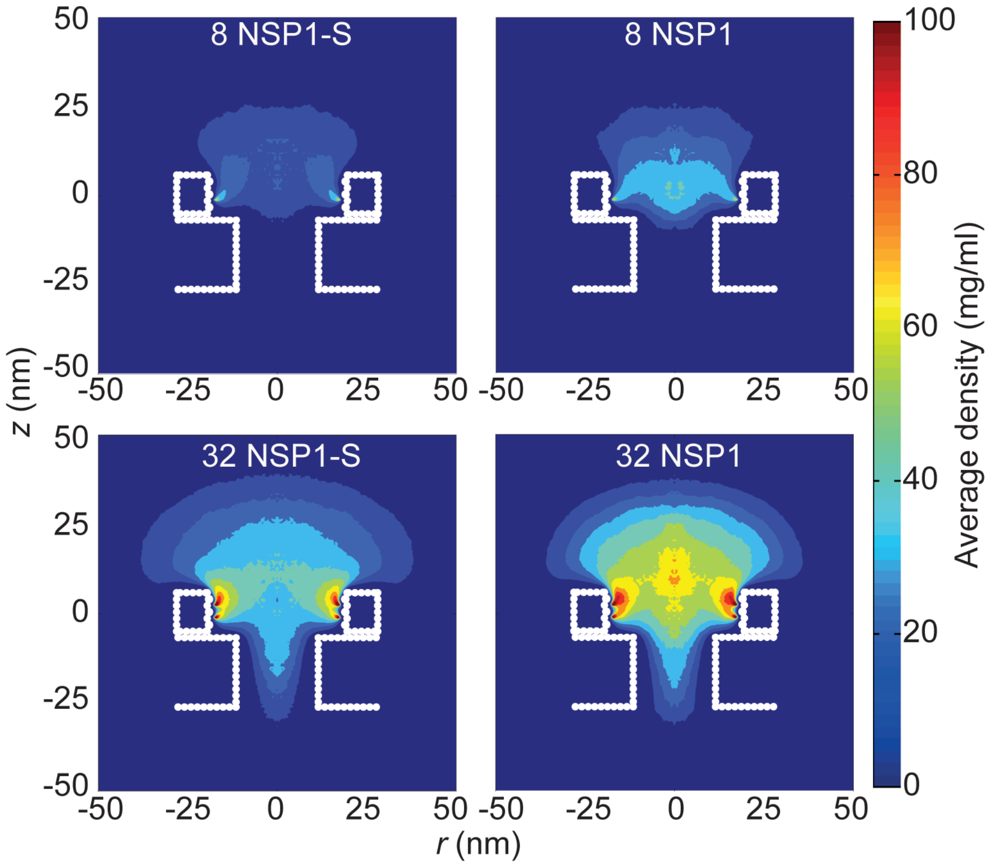

Supplementary Figure 15. Time-averaged *r-z* density distribution of rings with 8 (top row) or 32 (bottom row) copies of NSP1 (right column) or NSP1-S (left column) docked on a nanopore. This data shows denser structures of NSP1 inside the nanopores compared to NSP1-S for the same number of copies.

Sequence	Description
TCACGCGGCATCACCTGAACCCCAAAAACAGGAA	core
TGAGAAACAATTTCTGTTTATCAGACGAT	core
AGGAGCGTGCCCAAAACAGCGTTAATATTTTGT	core
TCACGGGGTCATAGCTAGGGAGC	core
GGCGGGTATTTATGTCTGGCATAACCCTCTTAATAAAACGAACTAAC	core
GTTAACTGCGCGTAACCACCACAC	core
AATGCGACATCCCATTACCCCGTTTT	core
TTGCTTTGACGAATAACATTATCCGAACGGGT	core
GGTGCCGGGGCGCTAGGGCGCTGGC	core
TTTAGGCTACCGACCGTGTGATCATGAG	core
ATTTTTGAAAAACGTGCAT	core
TTTGCACGAGAAAGGCCGGAGTTTCGGTCA	core
AGAAAAGCAAAGTCAGAGGAGCATGTCA	core
TAGTAAATATCAACGTAACAAAGCTGCTCATTCAGTGAATAAGG	core
CGAGCATGTGCGGGAACCGTTCTAGCTGAACAGAATCAACCGCCTCC	core
CAGAATGGAAAGCGCAGAGGGAGTTATTTAATGGGAAACAAGCGAAT	core
AGGTGAAAAAATGGTGTCCGCCGCGCTT	core
GCCACGAATGCGGGTTAAAGTGTAGCGG	core
GCATCAGCACATCAGACAAGAAAGCGAA	core
ATTCCAAGGTATTCTTGAGAGATCTACAA	core
TTCACGTTCTGACCAACTTTGAAAGAGGACAGATGAACGGTGTA	core
CAAGCAAGGCGCCCGAGCAAACAAGAGAA	core
ATCGGTTTATCCATGTTACTTAGCCGGAACGAGGCGCAGACGGTC	core
TAATACAACAGAAATAAAGAAATTCCGTC	core
TTAGAGGCGTAGATTAATTTTCCCTTAGGTCTGAGGGCGCATAAAATTC	core
ATACAGATATCTCAATATTAGAACCTCTGTG	core
TATTCACGTCTCGTCGGTCGCTCAGCATCC	core
TCAGGAGGTTTAGTACCGCCACCCTCAGAACCGCCACGTAACGAT	core
CATCATTTGCGGCGCGGTCTATCGTTAGTAGCACGTATAACG	core
AATATATTATTAGGAGCAGAAGATTGGTGCC	core
TGAATAATGGAAATTCGATAACACCCCTAA	core
CACGTAAATTTGAAGCAAATGAATGG	core
ATAGTTGCGACAACGGAGATTTGTATCATCGCCTGATAAATTGTG	core
CAAAAGAAGATGATTTTGAAAAGAGGCAAACGCGC	core
CCGCCAGAAATAAGCGCCAACAGATAAG	core
ATAGCTCATATGCGTTATACATCGGTGCGTCCGTGGAATTAT	core
GAGGTGGAGCCGCCACGGGACGTTGTAAGATCGC	core
TATATGTAACACTGAGTTTCGTCACCAATAGGA	core
GCAAGACATAATGGAAACAGTACATAAAATGCAAA	core
CGAGAAACGAGGGTAGCAACGGCTACAGAGGCTTTCATTATACCAGTC	core
ACTTTTTCTTTCATTGATTGCTTTGAA	core
ACCTAAAAAGGCCGCTTTTGCGGGATCGTCACC	core
GCGTTAACATCGCCCACGCATAACCGATATATT	core
GCCTGTTTGAATTTCTTAAACAGCTTGATACCG	core
TAGTATCTCACGGAAAAAGAGACGCAGTAGAAAAACTCAAC	core
CGCTATTAGGCTCCAAAAGGAGCCTTTAATTGT	core
ACGCCAGTTTGTGAGAGATAGACTTTCGGCCTCTTCTGTTG	core
CTGGCGAATTCGCCACAAGTACCCAGATAT	core
TTAAGTTACTAAAGGAATTGCGAATAATAATTTT	core
GGGTAACAATCGGCGAAACGTACAGCGCAAGGCGAGGAAAC	core
AACGACGTTTTGCTAAACAACTTTCAACAGTTT	core
GCCAGTGCCAAGCACCGACAGTGATTGTATAATTAAC	core
TTTATCACTAAAGTTTTGTCGTCTTTCCAGACGT	core
AAATCATAGAATCCTTGAAAACATAGCATAGTGAACTGCCAATTGTAAA	core
CGGTCGCTGCACTAAAACACTCATCTTTGACCCCCAGCGATTATA	core
CTCAGCAGCATACGTAATGCCACTACGAAGGCACCAACCTAAAAC	core
GGATTCTAATTCGCCCCAATCCAAATAAGAAACGAAACGCCA	core
TGCCGGAGAGGGTAGCTATTTTAAGAAC	core
AAGTACCTTTTAGTTAATTTCAAAATTAATTACATTTAACAAAAATATA	core
CTTGCCCTGACGAGAAACATAGTTAGCCCTCAGAAATGTTTAG	core
CTTAATTGGAATCATAATTACAAACAGCCCCGTA	core
AGCGCCAAGGGGGATGTGCTGCCATGTTTTCATC	core
TCCGGCAATATGTACCCCGGTTACCATAAATGGCGGATAA	core
CCAGCTTTTTCCCAGTCACGAACGGATA	core
GAAGGATTAGGATTAGCGGGGTTTTGCAAAGGAACA	core
GGGACGAGCTGAGAAGAGTCAGATAGCTTATTTG	core
CGGAACCTATTATTCTGAAACATGAAACCAAAAAAA	core
CAATAGGTTTTTTGAGTAAGCAGATAGCCGAACAAAGTTACCA	core
ATATAAAAGCTATATTTTCATTTGGGGGACGACGAT	core
AGACGACCAGATACATTGAGATTTAGGAATACCACAATTAAAGC	core
ACAACATTAACGAGAAATGCAATGCCTG	core
TTTAATTTCAACTTTAATCATTGTGAATTA	core
AAGACTCCTTATTACGCATAGTAGCAT	core
ATAATATTAAATCAAGATTAGTTGCTATTCCTGAA	core
TTCATCAGTAACGCCAAATAAATCATACAGGCAAGGCAAAGAAT	core
TTATTTCAATAAGCAATAAAGCCTCAGATATTCACA	core
CCTTATCGGAAGGGCGAAATTCTTACCATAATCGGC	core
GAGAAGCCTAGTAATGTGTAGGTAAAGATTC	core
TAGCCCACATTATGACCCTGTAATACTTTTGCGG	core
ACTGGATAGCGTCCAATACTGCGGAATCCATATAAAAAAGGGC	core
CGGATATTCATTACCCAAGAATTTTCATAGGTGTAGAATCCCC	core
ACCCATGTACCGGAAGAAAAATCTACGGTTTACCACGCGAGCTG	core
GGAACAACATTATTACAGGTAGAAAGAGCATCGGAA	core
AGGACGTTGGTAAATGCTGTCAATATTACCTTTTACATC	core
CAGACCAGGCGCATAGGCGAGAATAGTCAGTACCACAAAAATC	core
TACAAAATTTCATCAAAGTAATTCTGTCCCCTAATTTGCCAGT	core
TATCCTGAATCTTACCAACGCGTTCAGC	core
AATCATAAGGGAACCGAAGAAAATCTGTATTAAGAATCAAAAA	core
GCGAGGCTGTCTTTGTTTTAATTAATGCCCCCTGCCTATTT	core
AGAAGGCTCACTTGCCTGGGCTATGG	core
AGCAAATGCACTCAGCGGATTGCGGCTGAGACTCCTCAAGA	core
TCGAAATCCGCGACCTGCTCAGCTTGTAAACAGTTCGTCAGAA	core
CCAAGCGCGAAACAAAGTCCGACAATGTGTACTGGACCACCAG	core
ACGGGAGAAGCAAAATATTCATTTCACCGTAC	core
GGAAGCCCCAATAATAAGAGCGCCAAAGACCAGTTGATT	core
GAAAGAGGCAAAAGAATAAGGCTTGCTCTCTGAATTGGCCTTG	core
GTGCCGTCGAGAGGGTTGATATAAGTATAGCCCGGATGTATGGGA	core
GAAGGAAGAAAATACATACATAAAGGTGGCAAC	core
CTTTTTCATGAGGAAGTTTCCATTAAACGGGTAAAGAAAGACA	core
CCTTATGCGATTTTAAGAACTGGCTGAGGACTAAAGA	core
GATATTCAGGTTTTGAAGCCTCCCATCCACCAGAACCTAATAAGTT	core
CCTGTTTAGAAACGCAAAGACACCACTTAAGAAA	core
CAGGTCATTGCCTGAGAGTCTGAATAGCA	core
ACGGTAATCGTAAAACTGTAATTGAGCGCTAATATCATGACGGAAA	core
AACCCACAAGAATTGAGTTAAGGCATTAG	core
TAACATCCAAAGGAATTACGAGGCATAGTAAGAGC	core
TAGCAAAATCGCAAGGATAAAAATTTTTAGAACTGGCATGATT	core
CCGCCACCCTCAGAGCCACCACCCTCAGAGCCGCCTAATTTAAGTAGGG	core
AACAAATAAATCCTCTTCAACTAATGGACAATAAAATATA	core
CTCAAATGCTTTAAACAGTTCAGAAAGTAGCTCAATTATTCAT	core
TTACCGTTCCAGTAAGCGTCATACATGGCTTTTGAT	core
CATCGGCATACAGTCAAATCACCATCAATAT	core
AGTTTGCCTTTAGCGTCAGACTGTAGCGCGTTTT	core
AGCATAAAGCTAAATCGGTTGTACCAAAACCTTATT	core
GCCGGAAACACTCCAACAGGTCAGGATTAGAGAGTA	core
TTTGGGAATTCATTTTTGCGGATGGCTTAGAGCTTA	core
TAAAGGTGAATTATCACCGTCACCGACTTGAGCCATCGATGA	core
GACATTCAACCGATTGAGGGAGGGAAGGTAAATATGAGAGAT	core
TCATATGGTTTACCAGCAAGAAACA	core

Sequence	Description
CCTTTAATTGCTCCTTTTAGAAGCAAATCGAGAACAGGCAA	core
AAAAGGTGGCATCAATTCTACTAATAGGTATGTTA	core
AACACTATCCTTCCTGTGTAACAACCCGTCTCCAATC	core
CAGCATTGACAGGAGGTTGAGGCAGGTCAACAATATGTAA	core
CATGTTTTAAATATGCAACTAAAGTACGGTGTCTGG	core
AGCGTTTGCCATCTTTTCAGCCGCCGC	core
ATAATCAAAATCACCGGAACCAGAGCCACCACCGGA	core
CTCAGAGCCGCCACCCAGCTTCAAAGCGAACCAGACAAATATCGC	core
CCCAATTCTGCGAACGAGTAGATTTAGT	core
ATTGCTGAATATAATGCTACGAGAATGGATAATCACTCCAG	core
TTAACGGGGTCAGTGCCTTGAGTAACAGTGCCCGTACTTTCGAGG	core
AAGTTTCATTCGTCATAATATTTAAGTTTGAG	core
GATACAGGAGACAACAACATAAGAATTTTCTGCTCATTTG	core
AAAAACCAAAATAGCGAGAGGCTTTTTTTTTAACTGACCG	core
CTCAATTTAGGAGCACTAACAACTAATAGA	core
GCAAAAGAAGTTTTGCCAGAGGGGGTATTTTTGTTAAATCAGCTCAT	core
TTGACCATTAGATACATTTCGCAAATGGTCAATAA	core
GGAATAAGTTTATTTTGTCACAATCAATAGAAAAT	core
ATGAAATAGCAATAGCTATCTTACCGAAGCCCTTT	core
CTTTACAGCATTAAAATAGTAAACCGCCACCCTCAGAGCCACCACCCTCATTTTCAG	core
GGATAGCAAGCCCAGTACAAACTACAACGCCTGTAGCATTCCACAG	core
TTTAACTGTGCACCTTCTGACCTGAAAGCGTAAGAA	core
TAATGGGATAGGTCACGTTGGTGTAGATGAGACTACCT	core
TTTTAACCTCCGGCTTAGGTTGGGTTCCTTGCATGAAT	core
ATTTTCAGGTTTAACGTCAGTTCTGTAAATCGTCGCTATTA	core
AAGCCGGCGAACGTGGCGAGAAAG	core
GAAGGGGATCCAGCGCAGTGTCACTGCGCGCC	core
GTCAAAAATGAAAATAGCAGC	core
TACGTGCCTTGCTGAACCTCAAATATCAAACC	core
ACAGCCCTCACCAGAACGAGTAGTAAATTGGGCTTGAGATGG	core
AAAAAAGGTAAAGGGTCAACCGCCATCACGCGGGAGGCTCACTGCCCG	core
CTTTCCTTAGAATCAGAGCAAATTCCCGACT	core
AAGCATCCTCACAGTTGA	core
GAGGTGCAGGAACGAGTAGCCGATCTCACATTA	core
CACACACCGGGTACCGACAGCACTCCAGAG	core
ATTGCGTTGCCTAAACAGGAG	core
GCCTGGGGTGAACGGTACGCCAGACTAAAGTGTAAA	core
AGCTAATAAAGGGATTTT	core
TGAGGAAAATCACATTCTGAATGCCCCCGAAATCGGAACCCT	core
CCTCATGGTCAGAGATTCTTCTGCGCTCGACGCTCACAATTC	core
TTTTGAGGTGCCGTAAAGCACTATTTAGAGCTTGACGGGGA	core
GCAACCAAATACTATAATAGACAGGCCTAATGAGTG	core
AAGTTTCCTGTGTGAAATTGTTATCATTCGT	core
GAATGCCGGATTACTGTTCTGGATCCACATACGAGCCGG	core
GAGAGAATAACATACCTGCAGACAAT	core
TAATACAACAGAAATAAAGAAATTCCGTC	core
ATTTTTGAAAAACGTGCAT	core
TCGTAACTCTAAAAATAGA	core
GCCCTTCACCGCCTGGCCCTGAGAGAGTTGCAGCA	loop
TTGGAACAAGAGTCCACTATTAAAGAACGTGGACTCCAACGT	loop
TTTGATGGTGGTTCCGAAATCGGCAAAATCCCTT	loop
ATAAATCAAAAGAATAGCCCGAGATAGGGTTGAGTGTTGTT	loop
AGCGGTCCACGCTGGTTTGCCCCAGCAGGCGAAAATCCTG	loop
CAAAGGGCGAAAAACCGTCTATCAGGGCGATGGCC	loop
TTTCTTTTCACCAGTGAGACGGGCAACAGCTGATT	loop
GAGAGGCGGTTTGCGTATTGGGCGCCAGGGTGGTT	loop
TCGTGCCAGCTGCATTAATGAATCGGCCAACGCGCGGG	loop
CACTACGTGAACCATCACCCAAATCAAGTTTTTTGGGG	loop
AATCATTCATACCGGGGGTACCAGTTTCCCCCAACAGGATTAGCAGAGCGAGG	protein attach YES
TTGAGTGGGTCAAAAGTAGAAGATTTCCCCCAACAGGATTAGCAGAGCGAGG	protein attach YES
CAGTGAGGCCACAAACGTTTCCCCCAACAGGATTAGCAGAGCGAGG	protein attach YES
ATCATCGTAGGAATCTTACACTACCGTTTCCCCCAACAGGATTAGCAGAGCGAGG	protein attach YES
TGAACACGATGCCGCGAATTTCCCCCAACAGGATTAGCAGAGCGAGG	protein attach YES
TCGCGTCCGTGAATCGTTTTCCCCCAACAGGATTAGCAGAGCGAGG	protein attach YES
GTGCTGCGGCCAGGCCATTTCCCCCAACAGGATTAGCAGAGCGAGG	protein attach YES
GGGGGTTTCATGGAGCTTATTAACAA	protein attach YES
GGGGGTTTGAAATAACGGCGCCAGTA	protein attach YES
GGGGGTTTCGAACCACCCTTCATAACCGCTACAGGGCGCGTA	protein attach YES
GGGGGTTTTCAAATGCGGTTGCGCAA	protein attach YES
GGGGGTTTAGAGACTGGTCAACAAAC	protein attach YES
GGGGGTTTACACGGTGGTGCTTAAATG	protein attach YES
GGGGGTTTGATAGCGCCTGCTCAGGAA	protein attach YES
GTTTTAGCGAACCTAACCGTCAGAATTTCCCCCAACAGGATTAGCAGAGCGAGG	protein attach YES
GGGGGTTTATATTTTGCAGGCCAACG	protein attach YES
GACAAAAGGTAACATGGTCTAACGGA	protein attach NO
TAATGCAGTTTTCGAAGCACCGTCGCT	protein attach NO
ATCGGCCAACAGTACTTTA	protein attach NO
CCGGCAGTTGTGTACATCGACATACTGATT	protein attach NO
CAAGAAAAGCCATATCGGCTGGCCGTT	protein attach NO
TAGAAACCAATCAAGTATAAAGCGCTTTTAGTG	protein attach NO
CGAACGATCCTGATTGTTTGGATTAAATCC	protein attach NO
GGCAGCGCAGAGACATCAAGAAAACATCTTCTGATAAGAG	protein attach NO
TCAAAAATCCGTGGGAGTTGGCAGGTTA	protein attach NO
ATTAAACTTCAGGCATGAGCCAACATT	protein attach NO
ATCATTATTCCTGATTATCAGATGA	protein attach NO
TCTAAATAACAGATGTGAGTGAATAAATATAACGGCGGAT	protein attach NO
TATTTTCCCGCTTCAAAACAGTTTGCC	protein attach NO
ACGTGCTTCGCCTTGAATTACCTTTTTAAGAACGTGAGCGAAGCCAGC	protein attach NO
ATGAAGGCCGCACAGGCGGCAGAAGGAGCGGTGAAGGG	protein attach NO
CAAACAGGGTTAGAACCTACCATATATTAG	protein attach NO
GTATTATACTTCACCTCACCGGAAACGCCAGGGT	protein attach NO
CAAAAGAACCCTCATATATTTTCGTCTTGCGTGCTTTACA	protein attach NO
TTTCTTTGCTCGGGTAAT	protein attach NO
AGAAGTCAAAATTAGATTAAGTTTCA	protein attach NO
TGCTTATACTTCTTTGAGCCTTG	protein attach NO
CGATGAAAAAATGGATCAAACTTAAAAAACACCGAGAATC	protein attach NO
GAAACCACCCTTCGCACTAATATCTGTAGCATCCTCGAGTCGGGAAACC	protein attach NO
CCTGCAGCCAGCCTATTA	protein attach NO
AAAGTTGGCAATACCAGTCCCGGAA	protein attach NO
AAAGAGTCTGTCCAGCCAT	protein attach NO
TTTTTTTTCAATTACCGTTGGGCGAACGCG	protein attach NO
GGAATGGGAGAAACAATAACGGACGGACTTGTAAA	protein attach NO
CACATTACCAAGTTACAAAATCGCCTCCGG	protein attach NO
GTTCAGCAAATCAACATC	protein attach NO
GTCTTTCTGAGAGCCAGCGGATTT	protein attach NO
GGGTACTGTTGCCCTGCATTTTA	protein attach NO
AGGGAACAGTTGAGAACGTCAGCACCAGT	protein attach NO
CTGGTCAATCCGCCGGGAACAAA	protein attach NO
AAAAGGGTCCAGCTGTGTTTTCTACAT	protein attach NO
GCCATTGGCGGTCAGTATCAACTC	protein attach NO
GCAAACGTAACCGAGGACCATATCCGTTTAATAAA	protein attach NO
TTGGCCAGCAACCGCAACCAGAG	protein attach NO
TGCAATCCAGCATCAGCGGTTAAA	protein attach NO
TTTGAGGTGCCATCCCACGTCCGT	protein attach NO

Supplementary Table 1. Sequences of DNA origami ring with 8 protein attachment anchors.

Sequence	Description
TCACGCGGCATCACCTGAACCCCAAAAACAGGAA	core
TGAGAAACAATTTCTGTTTATCAGACGAT	core
AGGAGCGTGCCCAAAACAGCGTTAATATTTTGT	core
TCACGGGGTCATAGCTAGGGAGC	core
GGCGGGTATTTATGTCTGGCATAACCCTCTTAATAAAACGAACTAAC	core
GTTAACTGCGCGTAACCACCACAC	core
AATGCGACATCCCATTACCCCGTTTT	core
TTGCTTTGACGAATAACATTATCCGAACGGGT	core
GGTGCCGGGGCGCTAGGGCGCTGGC	core
TTTAGGCTACCGACCGTGTGATCATGAG	core
ATTTTTGAAAAACGTGCAT	core
TTTGCACGAGAAAGGCCGGAGTTTCGGTCA	core
AGAAAAGCAAAGTCAGAGGAGCATGTCA	core
TAGTAAATATCAACGTAACAAAGCTGCTCATTCAGTGAATAAGG	core
CGAGCATGTGCGGGAACCGTTCTAGCTGAACAGAATCAACCGCCTCC	core
CAGAATGGAAAGCGCAGAGGGAGTTATTTAATGGGAAACAAGCGAAT	core
AGGTGAAAAAATGGTGTCCGCCGCGCTT	core
GCCACGAATGCGGGTTAAAGTGTAGCGG	core
GCATCAGCACATCAGACAAGAAAGCGAA	core
ATTCCAAGGTATTCTTGAGAGATCTACAA	core
TTCACGTTCTGACCAACTTTGAAAGAGGACAGATGAACGGTGTA	core
CAAGCAAGGCGCCCGAGCAAACAAGAGAA	core
ATCGGTTTATCCATGTTACTTAGCCGGAACGAGGCGCAGACGGTC	core
TAATACAACAGAAATAAAGAAATTCCGTC	core
TTAGAGGCGTAGATTAATTTTCCCTTAGGTCTGAGGGCGCATAAAATTC	core
ATACAGATATCTCAATATTAGAACCTCTGTG	core
TATTCACGTCTCGTCGGTCGCTCAGCATCC	core
TCAGGAGGTTTAGTACCGCCACCCTCAGAACCGCCACGTAACGAT	core
CATCATTTGCGGCGCGGTCTATCGTTAGTAGCACGTATAACG	core
AATATATTATTAGGAGCAGAAGATTGGTGCC	core
TGAATAATGGAAATTCGATAACACCCCTAA	core
CACGTAAATTTGAAGCAAATGAATGG	core
ATAGTTGCGACAACGGAGATTTGTATCATCGCCTGATAAATTGTG	core
CAAAAGAAGATGATTTTGAAAAGAGGCAAACGCGC	core
CCGCCAGAAATAAGCGCCAACAGATAAG	core
ATAGCTCATATGCGTTATACATCGGTGCGTCCGTGGAATTAT	core
GAGGTGGAGCCGCCACGGGACGTTGTAAGATCGC	core
TATATGTAACACTGAGTTTCGTCACCAATAGGA	core
GCAAGACATAATGGAAACAGTACATAAAATGCAAA	core
CGAGAAACGAGGGTAGCAACGGCTACAGAGGCTTTCATTATACCAGTC	core
ACTTTTTCTTTCATTGATTGCTTTGAA	core
ACCTAAAAAGGCCGCTTTTGCGGGATCGTCACC	core
GCGTTAACATCGCCCACGCATAACCGATATATT	core
GCCTGTTTGAATTTCTTAAACAGCTTGATACCG	core
TAGTATCTCACGGAAAAAGAGACGCAGTAGAAAAACTCAAC	core
CGCTATTAGGCTCCAAAAGGAGCCTTTAATTGT	core
ACGCCAGTTTGTGAGAGATAGACTTTCGGCCTCTTCTGTTG	core
CTGGCGAATTCGCCACAAGTACCCAGATAT	core
TTAAGTTACTAAAGGAATTGCGAATAATAATTTT	core
GGGTAACAATCGGCGAAACGTACAGCGCAAGGCGAGGAAAC	core
AACGACGTTTTGCTAAACAACTTTCAACAGTTT	core
GCCAGTGCCAAGCACCGACAGTGATTGTATAATTAAC	core
TTTATCACTAAAGTTTTGTCGTCTTTCCAGACGT	core
AAATCATAGAATCCTTGAAAACATAGCATAGTGAACTGCCAATTGTAAA	core
CGGTCGCTGCACTAAAACACTCATCTTTGACCCCCAGCGATTATA	core
CTCAGCAGCATACGTAATGCCACTACGAAGGCACCAACCTAAAAC	core
GGATTCTAATTCGCCCCAATCCAAATAAGAAACGAAACGCCA	core
TGCCGGAGAGGGTAGCTATTTTAAGAAC	core
AAGTACCTTTTAGTTAATTTCAAAATTAATTACATTTAACAAAAATATA	core
CTTGCCCTGACGAGAAACATAGTTAGCCCTCAGAAATGTTTAG	core
CTTAATTGGAATCATAATTACAAACAGCCCCGTA	core
AGCGCCAAGGGGGATGTGCTGCCATGTTTTCATC	core
TCCGGCAATATGTACCCCGGTTACCATAAATGGCGGATAA	core
CCAGCTTTTTCCCAGTCACGAACGGATA	core
GAAGGATTAGGATTAGCGGGGTTTTGCAAAGGAACA	core
GGGACGAGCTGAGAAGAGTCAGATAGCTTATTTG	core
CGGAACCTATTATTCTGAAACATGAAACCAAAAAAA	core
CAATAGGTTTTTTGAGTAAGCAGATAGCCGAACAAAGTTACCA	core
ATATAAAAGCTATATTTTCATTTGGGGGACGACGAT	core
AGACGACCAGATACATTGAGATTTAGGAATACCACAATTAAAGC	core
ACAACATTAACGAGAAATGCAATGCCTG	core
TTTAATTTCAACTTTAATCATTGTGAATTA	core
AAGACTCCTTATTACGCATAGTAGCAT	core
ATAATATTAAATCAAGATTAGTTGCTATTCCTGAA	core
TTCATCAGTAACGCCAAATAAATCATACAGGCAAGGCAAAGAAT	core
TTATTTCAATAAGCAATAAAGCCTCAGATATTCACA	core
CCTTATCGGAAGGGCGAAATTCTTACCATAATCGGC	core
GAGAAGCCTAGTAATGTGTAGGTAAAGATTC	core
TAGCCCACATTATGACCCTGTAATACTTTTGCGG	core
ACTGGATAGCGTCCAATACTGCGGAATCCATATAAAAAAGGGC	core
CGGATATTCATTACCCAAGAATTTTCATAGGTGTAGAATCCCC	core
ACCCATGTACCGGAAGAAAAATCTACGGTTTACCACGCGAGCTG	core
GGAACAACATTATTACAGGTAGAAAGAGCATCGGAA	core
AGGACGTTGGTAAATGCTGTCAATATTACCTTTTACATC	core
CAGACCAGGCGCATAGGCGAGAATAGTCAGTACCACAAAAATC	core
TACAAAATTTCATCAAAGTAATTCTGTCCCCTAATTTGCCAGT	core
TATCCTGAATCTTACCAACGCGTTCAGC	core
AATCATAAGGGAACCGAAGAAAATCTGTATTAAGAATCAAAAA	core
GCGAGGCTGTCTTTGTTTTAATTAATGCCCCCTGCCTATTT	core
AGAAGGCTCACTTGCCTGGGCTATGG	core
AGCAAATGCACTCAGCGGATTGCGGCTGAGACTCCTCAAGA	core
TCGAAATCCGCGACCTGCTCAGCTTGTAAACAGTTCGTCAGAA	core
CCAAGCGCGAAACAAAGTCCGACAATGTGTACTGGACCACCAG	core
ACGGGAGAAGCAAAATATTCATTTCACCGTAC	core
GGAAGCCCCAATAATAAGAGCGCCAAAGACCAGTTGATT	core
GAAAGAGGCAAAAGAATAAGGCTTGCTCTCTGAATTGGCCTTG	core
GTGCCGTCGAGAGGGTTGATATAAGTATAGCCCGGATGTATGGGA	core
GAAGGAAGAAAATACATACATAAAGGTGGCAAC	core
CTTTTTCATGAGGAAGTTTCCATTAAACGGGTAAAGAAAGACA	core
CCTTATGCGATTTTAAGAACTGGCTGAGGACTAAAGA	core
GATATTCAGGTTTTGAAGCCTCCCATCCACCAGAACCTAATAAGTT	core
CCTGTTTAGAAACGCAAAGACACCACTTAAGAAA	core
CAGGTCATTGCCTGAGAGTCTGAATAGCA	core
ACGGTAATCGTAAAACTGTAATTGAGCGCTAATATCATGACGGAAA	core
AACCCACAAGAATTGAGTTAAGGCATTAG	core
TAACATCCAAAGGAATTACGAGGCATAGTAAGAGC	core
TAGCAAAATCGCAAGGATAAAAATTTTTAGAACTGGCATGATT	core
CCGCCACCCTCAGAGCCACCACCCTCAGAGCCGCCTAATTTAAGTAGGG	core
AACAAATAAATCCTCTTCAACTAATGGACAATAAAATATA	core
CTCAAATGCTTTAAACAGTTCAGAAAGTAGCTCAATTATTCAT	core
TTACCGTTCCAGTAAGCGTCATACATGGCTTTTGAT	core
CATCGGCATACAGTCAAATCACCATCAATAT	core
AGTTTGCCTTTAGCGTCAGACTGTAGCGCGTTTT	core
AGCATAAAGCTAAATCGGTTGTACCAAAACCTTATT	core
GCCGGAAACACTCCAACAGGTCAGGATTAGAGAGTA	core
TTTGGGAATTCATTTTTGCGGATGGCTTAGAGCTTA	core
TAAAGGTGAATTATCACCGTCACCGACTTGAGCCATCGATGA	core
GACATTCAACCGATTGAGGGAGGGAAGGTAAATATGAGAGAT	core
TCATATGGTTTACCAGCAAGAAACA	core

Sequence	Description
CCTTTAATTGCTCCTTTTAGAAGCAAATCGAGAACAGGCAA	core
AAAAGGTGGCATCAATTCTACTAATAGGTATGTTA	core
AACACTATCCTTCCTGTGTAACAACCCGTCTCCAATC	core
CAGCATTGACAGGAGGTTGAGGCAGGTCAACAATATGTAA	core
CATGTTTTAAATATGCAACTAAAGTACGGTGTCTGG	core
AGCGTTTGCCATCTTTTCAGCCGCCGC	core
ATAATCAAAATCACCGGAACCAGAGCCACCACCGGA	core
CTCAGAGCCGCCACCCAGCTTCAAAGCGAACCAGACAAATATCGC	core
CCCAATTCTGCGAACGAGTAGATTTAGT	core
ATTGCTGAATATAATGCTACGAGAATGGATAATCACTCCAG	core
TTAACGGGGTCAGTGCCTTGAGTAACAGTGCCCGTACTTTCGAGG	core
AAGTTTCATTCGTCATAATATTTAAGTTTGAG	core
GATACAGGAGACAACAACATAAGAATTTTCTGCTCATTTG	core
AAAAACCAAAATAGCGAGAGGCTTTTTTTTTAACTGACCG	core
CTCAATTTAGGAGCACTAACAACTAATAGA	core
GCAAAAGAAGTTTTGCCAGAGGGGGTATTTTTGTTAAATCAGCTCAT	core
TTGACCATTAGATACATTTCGCAAATGGTCAATAA	core
GGAATAAGTTTATTTTGTCACAATCAATAGAAAAT	core
ATGAAATAGCAATAGCTATCTTACCGAAGCCCTTT	core
CTTTACAGCATTAAAATAGTAAACCGCCACCCTCAGAGCCACCACCCTCATTTTCAG	core
GGATAGCAAGCCCAGTACAAACTACAACGCCTGTAGCATTCCACAG	core
TTTAACTGTGCACCTTCTGACCTGAAAGCGTAAGAA	core
TAATGGGATAGGTCACGTTGGTGTAGATGAGACTACCT	core
TTTTAACCTCCGGCTTAGGTTGGGTTCCTTGCATGAAT	core
ATTTTCAGGTTTAACGTCAGTTCTGTAAATCGTCGCTATTA	core
AAGCCGGCGAACGTGGCGAGAAAG	core
GAAGGGGATCCAGCGCAGTGTCACTGCGCGCC	core
GTCAAAAATGAAAATAGCAGC	core
TACGTGCCTTGCTGAACCTCAAATATCAAACC	core
ACAGCCCTCACCAGAACGAGTAGTAAATTGGGCTTGAGATGG	core
AAAAAAGGTAAAGGGTCAACCGCCATCACGCGGGAGGCTCACTGCCCG	core
CTTTCCTTAGAATCAGAGCAAATTCCCGACT	core
AAGCATCCTCACAGTTGA	core
GAGGTGCAGGAACGAGTAGCCGATCTCACATTA	core
CACACACCGGGTACCGACAGCACTCCAGAG	core
ATTGCGTTGCCTAAACAGGAG	core
GCCTGGGGTGAACGGTACGCCAGACTAAAGTGTAAA	core
AGCTAATAAAGGGATTTT	core
TGAGGAAAATCACATTCTGAATGCCCCCGAAATCGGAACCCT	core
CCTCATGGTCAGAGATTCTTCTGCGCTCGACGCTCACAATTC	core
TTTTGAGGTGCCGTAAAGCACTATTTAGAGCTTGACGGGGA	core
GCAACCAAATACTATAATAGACAGGCCTAATGAGTG	core
AAGTTTCCTGTGTGAAATTGTTATCATTCGT	core
GAATGCCGGATTACTGTTCTGGATCCACATACGAGCCGG	core
GAGAGAATAACATACCTGCAGACAAT	core
TAATACAACAGAAATAAAGAAATTCCGTC	core
ATTTTTGAAAAACGTGCAT	core
TCGTAACTCTAAAAATAGA	core
GCCCTTCACCGCCTGGCCCTGAGAGAGTTGCAGCA	loop
TTGGAACAAGAGTCCACTATTAAAGAACGTGGACTCCAACGT	loop
TTTGATGGTGGTTCCGAAATCGGCAAAATCCCTT	loop
ATAAATCAAAAGAATAGCCCGAGATAGGGTTGAGTGTTGTT	loop
AGCGGTCCACGCTGGTTTGCCCCAGCAGGCGAAAATCCTG	loop
CAAAGGGCGAAAAACCGTCTATCAGGGCGATGGCC	loop
TTTCTTTTCACCAGTGAGACGGGCAACAGCTGATT	loop
GAGAGGCGGTTTGCGTATTGGGCGCCAGGGTGGTT	loop
TCGTGCCAGCTGCATTAATGAATCGGCCAACGCGCGGG	loop
CACTACGTGAACCATCACCCAAATCAAGTTTTTTGGGG	loop
AATCATTCATACCGGGGGTACCAGTTTCCCCCAACAGGATTAGCAGAGCGAGG	protein attach YES
TTGAGTGGGTCAAAAGTAGAAGATTTCCCCCAACAGGATTAGCAGAGCGAGG	protein attach YES
CAGTGAGGCCACAAACGTTTCCCCCAACAGGATTAGCAGAGCGAGG	protein attach YES
ATCATCGTAGGAATCTTACACTACCGTTTCCCCCAACAGGATTAGCAGAGCGAGG	protein attach YES
TGAACACGATGCCGCGAATTTCCCCCAACAGGATTAGCAGAGCGAGG	protein attach YES
TCGCGTCCGTGAATCGTTTTCCCCCAACAGGATTAGCAGAGCGAGG	protein attach YES
GTGCTGCGGCCAGGCCATTTCCCCCAACAGGATTAGCAGAGCGAGG	protein attach YES
GGGGGTTTCATGGAGCTTATTAACAA	protein attach YES
GGGGGTTTGAAATAACGGCGCCAGTA	protein attach YES
GGGGGTTTCGAACCACCCTTCATAACCGCTACAGGGCGCGTA	protein attach YES
GGGGGTTTTCAAATGCGGTTGCGCAA	protein attach YES
GGGGGTTTAGAGACTGGTCAACAAAC	protein attach YES
GGGGGTTTACACGGTGGTGCTTAAATG	protein attach YES
GGGGGTTTGATAGCGCCTGCTCAGGAA	protein attach YES
GTTTTAGCGAACCTAACCGTCAGAATTTCCCCCAACAGGATTAGCAGAGCGAGG	protein attach YES
GGGGGTTTATATTTTGCAGGCCAACG	protein attach YES
GACAAAAGGTAACATGGTCTAACGGTTTCCCCCAACAGGATTAGCAGAGCGAGG	protein attach YES
TAATGCAGTTTTCGAAGCACCGTCGCTTTCCCCCAACAGGATTAGCAGAGCGAGG	protein attach YES
ATCGGCCAACAGTACTTTTTTCCCCCAACAGGATTAGCAGAGCGAGG	protein attach YES
GGGGGTTTCGGCAGTTGTGTACATCGACATACTGATT	protein attach YES
CAAGAAAAGCCATATCGGCTGGCCGTTTTCCCCCAACAGGATTAGCAGAGCGAGG	protein attach YES
TAGAAACCAATCAAGTATAAAGCGCTTTAGTGTTTCCCCCAACAGGATTAGCAGAGCGAGG	protein attach YES
GGGGGTTTGAACGATCCTGATTGTTTGGATTAAATCC	protein attach YES
GGGGGTTTGCAGCGCAGAGACATCAAGAAAACATCTTCTGATAAGAG	protein attach YES
TCAAAAATCCGTGGGAGTTGGCAGGTTTTTCCCCCAACAGGATTAGCAGAGCGAGG	protein attach YES
ATTAAACTTCAGGCATGAGCCAACATTTTCCCCCAACAGGATTAGCAGAGCGAGG	protein attach YES
GGGGGTTTTCATTATTCCTGATTATCAGATGA	protein attach YES
GGGGGTTTCTAAATAACAGATGTGAGTGAATAAATATAACGGCGGAT	protein attach YES
TATTTTCCCGCTTCAAAACAGTTTGCTTTCCCCCAACAGGATTAGCAGAGCGAGG	protein attach YES
GGGGGTTTCGTGCTTCGCCTTGAATTACCTTTTTAAGAACGTGAGCGAAGCCAGC	protein attach YES
GGGGGTTTTGAAGGCCGCACAGGCGGCAGAAGGAGCGGTGAAGGG	protein attach YES
GGGGGTTTAAACAGGGTTAGAACCTACCATATATTAG	protein attach YES
GGGGGTTTTATTATACTTCACCTCACCGGAAACGCCAGGGT	protein attach YES
CAAAAGAACCCTCATATATTTTCGTCTTGCGTGTTTACATTTCCCCCAACAGGATTAGCAGAGCGAGG	protein attach YES
TTTCTTTGCTCGGGTAATTTCCCCCAACAGGATTAGCAGAGCGAGG	protein attach YES
GGGGGTTTGAAGTCAAAATTAGATTAAGTTTCA	protein attach YES
TGCTTATACTTCTTTGAGCCTTTTTCCCCCAACAGGATTAGCAGAGCGAGG	protein attach YES
GGGGGTTTGATGAAAAAATGGATCAAACTTAAAAAACACCGAGAATC	protein attach YES
GGGGGTTTAAACCACCCTTCGCACTAATATCTGTAGCATCCTCGAGTCGGGAAACCGGGGGTTT	protein attach YES
CCTGCAGCCAGCCTATTTTTCCCCCAACAGGATTAGCAGAGCGAGG	protein attach YES
GGGGGTTTAAGTTGGCAATACCAGTCCCGGAA	protein attach YES
AAAGAGTCTGTCCAGCCATTTCCCCCAACAGGATTAGCAGAGCGAGG	protein attach YES
GGGGGTTTTTTTTTTCAATTACCGTTGGGCGAACGCG	protein attach YES
GGGGGTTTGAATGGGAGAAACAATAACGGACGGACTTGTAATTTCCCCCAACAGGATTAGCAGAGCGAGG	protein attach YES
GGGGGTTTACATTACCAAGTTACAAAATCGCCTCCGG	protein attach YES
GTTCAGCAAATCAACATTTTCCCCCAACAGGATTAGCAGAGCGAGG	protein attach YES
GGGGGTTTTCTTTCTGAGAGCCAGCGGATTTTTCCCCCAACAGGATTAGCAGAGCGAGG	protein attach YES
GGGGGTTTGGTACTGTTGCCCTGCATTTTTTTCCCCCAACAGGATTAGCAGAGCGAGG	protein attach YES
GGGGGTTTGGGAACAGTTGAGAACGTCAGCACCAGT	protein attach YES
GGGGGTTTTGGTCAATCCGCCGGGAACAATTTCCCCCAACAGGATTAGCAGAGCGAGG	protein attach YES
AAAAGGGTCCAGCTGTGTTTTCTACATTTCCCCCAACAGGATTAGCAGAGCGAGG	protein attach YES
GGGGGTTTCCATTGGCGGTCAGTATCAACTTTTCCCCCAACAGGATTAGCAGAGCGAGG	protein attach YES
GCAAACGTAACCGAGGACCATATCCGTTTAATAATTTCCCCCAACAGGATTAGCAGAGCGAGG	protein attach YES
GGGGGTTTTGGCCAGCAACCGCAACCAGATTTCCCCCAACAGGATTAGCAGAGCGAGG	protein attach YES
GGGGGTTTGCAATCCAGCATCAGCGGTTAATTTCCCCCAACAGGATTAGCAGAGCGAGG	protein attach YES
GGGGGTTTTTGAGGTGCCATCCCACGTCCGTTTCCCCCAACAGGATTAGCAGAGCGAGG	protein attach YES

Supplementary Table 2. Sequences of DNA origami ring with 32 protein attachment anchors.

Sequence	Description
TCACGCGGCATCACCTGAACCCCAAAAACAGGAA	core
TGAGAAACAATTTCTGTTTATCAGACGAT	core
AGGAGCGTGCCCAAAACAGCGTTAATATTTTGT	core
TCACGGGGTCATAGCTAGGGAGC	core
GGCGGGTATTTATGTCTGGCATAACCCTCTTAATAAAACGAACTAAC	core
GTTAACTGCGCGTAACCACCACAC	core
AATGCGACATCCCATTACCCCGTTTT	core
TTGCTTTGACGAATAACATTATCCGAACGGGT	core
GGTGCCGGGGCGCTAGGGCGCTGGC	core
TTTAGGCTACCGACCGTGTGATCATGAG	core
ATTTTTGAAAAACGTGCAT	core
TTTGCACGAGAAAGGCCGGAGTTTCGGTCA	core
AGAAAAGCAAAGTCAGAGGAGCATGTCA	core
TAGTAAATATCAACGTAACAAAGCTGCTCATTCAGTGAATAAGG	core
CGAGCATGTGCGGGAACCGTTCTAGCTGAACAGAATCAACCGCCTCC	core
CAGAATGGAAAGCGCAGAGGGAGTTATTTAATGGGAAACAAGCGAAT	core
AGGTGAAAAAATGGTGTCCGCCGCGCTT	core
GCCACGAATGCGGGTTAAAGTGTAGCGG	core
GCATCAGCACATCAGACAAGAAAGCGAA	core
ATTCCAAGGTATTCTTGAGAGATCTACAA	core
TTCACGTTCTGACCAACTTTGAAAGAGGACAGATGAACGGTGTA	core
CAAGCAAGGCGCCCGAGCAAACAAGAGAA	core
ATCGGTTTATCCATGTTACTTAGCCGGAACGAGGCGCAGACGGTC	core
TAATACAACAGAAATAAAGAAATTCCGTC	core
TTAGAGGCGTAGATTAATTTTCCCTTAGGTCTGAGGGCGCATAAAATTC	core
ATACAGATATCTCAATATTAGAACCTCTGTG	core
TATTCACGTCTCGTCGGTCGCTCAGCATCC	core
TCAGGAGGTTTAGTACCGCCACCCTCAGAACCGCCACGTAACGAT	core
CATCATTTGCGGCGCGGTCTATCGTTAGTAGCACGTATAACG	core
AATATATTATTAGGAGCAGAAGATTGGTGCC	core
TGAATAATGGAAATTCGATAACACCCCTAA	core
CACGTAAATTTGAAGCAAATGAATGG	core
ATAGTTGCGACAACGGAGATTTGTATCATCGCCTGATAAATTGTG	core
CAAAAGAAGATGATTTTGAAAAGAGGCAAACGCGC	core
CCGCCAGAAATAAGCGCCAACAGATAAG	core
ATAGCTCATATGCGTTATACATCGGTGCGTCCGTGGAATTAT	core
GAGGTGGAGCCGCCACGGGACGTTGTAAGATCGC	core
TATATGTAACACTGAGTTTCGTCACCAATAGGA	core
GCAAGACATAATGGAAACAGTACATAAAATGCAAA	core
CGAGAAACGAGGGTAGCAACGGCTACAGAGGCTTTCATTATACCAGTC	core
ACTTTTTCTTTCATTGATTGCTTTGAA	core
ACCTAAAAAGGCCGCTTTTGCGGGATCGTCACC	core
GCGTTAACATCGCCCACGCATAACCGATATATT	core
GCCTGTTTGAATTTCTTAAACAGCTTGATACCG	core
TAGTATCTCACGGAAAAAGAGACGCAGTAGAAAAACTCAAC	core
CGCTATTAGGCTCCAAAAGGAGCCTTTAATTGT	core
ACGCCAGTTTGTGAGAGATAGACTTTCGGCCTCTTCTGTTG	core
CTGGCGAATTCGCCACAAGTACCCAGATAT	core
TTAAGTTACTAAAGGAATTGCGAATAATAATTTT	core
GGGTAACAATCGGCGAAACGTACAGCGCAAGGCGAGGAAAC	core
AACGACGTTTTGCTAAACAACTTTCAACAGTTT	core
GCCAGTGCCAAGCACCGACAGTGATTGTATAATTAAC	core
TTTATCACTAAAGTTTTGTCGTCTTTCCAGACGT	core
AAATCATAGAATCCTTGAAAACATAGCATAGTGAACTGCCAATTGTAAA	core
CGGTCGCTGCACTAAAACACTCATCTTTGACCCCCAGCGATTATA	core
CTCAGCAGCATACGTAATGCCACTACGAAGGCACCAACCTAAAAC	core
GGATTCTAATTCGCCCCAATCCAAATAAGAAACGAAACGCCA	core
TGCCGGAGAGGGTAGCTATTTTAAGAAC	core
AAGTACCTTTTAGTTAATTTCAAAATTAATTACATTTAACAAAAATATA	core
CTTGCCCTGACGAGAAACATAGTTAGCCCTCAGAAATGTTTAG	core
CTTAATTGGAATCATAATTACAAACAGCCCCGTA	core
AGCGCCAAGGGGGATGTGCTGCCATGTTTTCATC	core
TCCGGCAATATGTACCCCGGTTACCATAAATGGCGGATAA	core
CCAGCTTTTTCCCAGTCACGAACGGATA	core
GAAGGATTAGGATTAGCGGGGTTTTGCAAAGGAACA	core
GGGACGAGCTGAGAAGAGTCAGATAGCTTATTTG	core
CGGAACCTATTATTCTGAAACATGAAACCAAAAAAA	core
CAATAGGTTTTTTGAGTAAGCAGATAGCCGAACAAAGTTACCA	core
ATATAAAAGCTATATTTTCATTTGGGGGACGACGAT	core
AGACGACCAGATACATTGAGATTTAGGAATACCACAATTAAAGC	core
ACAACATTAACGAGAAATGCAATGCCTG	core
TTTAATTTCAACTTTAATCATTGTGAATTA	core
ATAATATTAAATCAAGATTAGTTGCTATTCCTGAA	core
TTCATCAGTAACGCCAAATAAATCATACAGGCAAGGCAAAGAAT	core
TTATTTCAATAAGCAATAAAGCCTCAGATATTCACA	core
CCTTATCGGAAGGGCGAAATTCTTACCATAATCGGC	core
CGGATATTCATTACCCAAGAATTTTCATAGGTGTAGAATCCCC	core
ACCCATGTACCGGAAGAAAAATCTACGGTTTACCACGCGAGCTG	core
GGAACAACATTATTACAGGTAGAAAGAGCATCGGAA	core
AGGACGTTGGTAAATGCTGTCAATATTACCTTTTACATC	core
CAGACCAGGCGCATAGGCGAGAATAGTCAGTACCACAAAAATC	core
TACAAAATTTCATCAAAGTAATTCTGTCCCCTAATTTGCCAGT	core
TATCCTGAATCTTACCAACGCGTTCAGC	core
AATCATAAGGGAACCGAAGAAAATCTGTATTAAGAATCAAAAA	core
GCGAGGCTGTCTTTGTTTTAATTAATGCCCCCTGCCTATTT	core
AGAAGGCTCACTTGCCTGGGCTATGG	core
AGCAAATGCACTCAGCGGATTGCGGCTGAGACTCCTCAAGA	core
TCGAAATCCGCGACCTGCTCAGCTTGTAAACAGTTCGTCAGAA	core
CCAAGCGCGAAACAAAGTCCGACAATGTGTACTGGACCACCAG	core
ACGGGAGAAGCAAAATATTCATTTCACCGTAC	core
GGAAGCCCCAATAATAAGAGCGCCAAAGACCAGTTGATT	core
GAAAGAGGCAAAAGAATAAGGCTTGCTCTCTGAATTGGCCTTG	core
GTGCCGTCGAGAGGGTTGATATAAGTATAGCCCGGATGTATGGGA	core
GAAGGAAGAAAATACATACATAAAGGTGGCAAC	core
CTTTTTCATGAGGAAGTTTCCATTAAACGGGTAAAGAAAGACA	core
CCTTATGCGATTTTAAGAACTGGCTGAGGACTAAAGA	core
GATATTCAGGTTTTGAAGCCTCCCATCCACCAGAACCTAATAAGTT	core
CCTGTTTAGAAACGCAAAGACACCACTTAAGAAA	core
CAGGTCATTGCCTGAGAGTCTGAATAGCA	core
ACGGTAATCGTAAAACTGTAATTGAGCGCTAATATCATGACGGAAA	core
AACCCACAAGAATTGAGTTAAGGCATTAG	core
TAACATCCAAAGGAATTACGAGGCATAGTAAGAGC	core
TAGCAAAATCGCAAGGATAAAAATTTTTAGAACTGGCATGATT	core
CCGCCACCCTCAGAGCCACCACCCTCAGAGCCGCCTAATTTAAGTAGGG	core
AACAAATAAATCCTCTTCAACTAATGGACAATAAAATATA	core
TTACCGTTCCAGTAAGCGTCATACATGGCTTTTGAT	core
AGCATAAAGCTAAATCGGTTGTACCAAAACCTTATT	core
GCCGGAAACACTCCAACAGGTCAGGATTAGAGAGTA	core
TTTGGGAATTCATTTTTGCGGATGGCTTAGAGCTTA	core
TCATATGGTTTACCAGCAAGAAACA	core
CCTTTAATTGCTCCTTTTAGAAGCAAATCGAGAACAGGCAA	core
AAAAGGTGGCATCAATTCTACTAATAGGTATGTTA	core
AACACTATCCTTCCTGTGTAACAACCCGTCTCCAATC	core
CAGCATTGACAGGAGGTTGAGGCAGGTCAACAATATGTAA	core
CATGTTTTAAATATGCAACTAAAGTACGGTGTCTGG	core
AGCGTTTGCCATCTTTTCAGCCGCCGC	core
ATAATCAAAATCACCGGAACCAGAGCCACCACCGGA	core
CTCAGAGCCGCCACCCAGCTTCAAAGCGAACCAGACAAATATCGC	core
CCCAATTCTGCGAACGAGTAGATTTAGT	core
ATTGCTGAATATAATGCTACGAGAATGGATAATCACTCCAG	core
TTAACGGGGTCAGTGCCTTGAGTAACAGTGCCCGTACTTTCGAGG	core
AAGTTTCATTCGTCATAATATTTAAGTTTGAG	core
GATACAGGAGACAACAACATAAGAATTTTCTGCTCATTTG	core
AAAAACCAAAATAGCGAGAGGCTTTTTTTTTAACTGACCG	core
CTCAATTTAGGAGCACTAACAACTAATAGA	core
GCAAAAGAAGTTTTGCCAGAGGGGGTATTTTTGTTAAATCAGCTCAT	core

Sequence	Description
TTGACCATTAGATACATTTCGCAAATGGTCAATAA	core
GGAATAAGTTTATTTTGTCACAATCAATAGAAAAT	core
ATGAAATAGCAATAGCTATCTTACCGAAGCCCTTT	core
CTTTACAGCATTAAAATAGTAAACCGCCACCCTCAGAGCCACCACCCTCATTTTCAG	core
GGATAGCAAGCCCAGTACAAACTACAACGCCTGTAGCATTCCACAG	core
TTTAACTGTGCACCTTCTGACCTGAAAGCGTAAGAA	core
TAATGGGATAGGTCACGTTGGTGTAGATGAGACTACCT	core
TTTTAACCTCCGGCTTAGGTTGGGTTCCTTGCATGAAT	core
ATTTTCAGGTTTAACGTCAGTTCTGTAAATCGTCGCTATTA	core
AAGCCGGCGAACGTGGCGAGAAAG	core
GAAGGGGATCCAGCGCAGTGTCACTGCGCGCC	core
GTCAAAAATGAAAATAGCAGC	core
TACGTGCCTTGCTGAACCTCAAATATCAAACC	core
ACAGCCCTCACCAGAACGAGTAGTAAATTGGGCTTGAGATGG	core
GAGAGAATAACATACCTGCAGACAAT	core
TAATACAACAGAAATAAAGAAATTCCGTC	core
ATTTTTGAAAAACGTGCAT	core
TCGTAACTCTAAAAATAGA	core
AAAAAAGGTAAAGGGTCAACCGCCATCACGCGGGAGGCTCACTGCCCG	core
CTTTCCTTAGAATCAGAGCAAATTCCCGACT	core
AAGCATCCTCACAGTTGA	core
GAGGTGCAGGAACGAGTAGCCGATCTCACATTA	core
CACACACCGGGTACCGACAGCACTCCAGAG	core
ATTGCGTTGCCTAAACAGGAG	core
GCCTGGGGTGAACGGTACGCCAGACTAAAGTGTAAA	core
AGCTAATAAAGGGATTTT	core
TGAGGAAAATCACATTCTGAATGCCCCCGAAATCGGAACCCT	core
CCTCATGGTCAGAGATTCTTCTGCGCTCGACGCTCACAATTC	core
TTTTGAGGTGCCGTAAAGCACTATTTAGAGCTTGACGGGGA	core
GCAACCAAATACTATAATAGACAGGCCTAATGAGTG	core
AAGTTTCCTGTGTGAAATTGTTATCATTCGT	core
GAATGCCGGATTACTGTTCTGGATCCACATACGAGCCGG	core
GCCCTTCACCGCCTGGCCCTGAGAGAGTTGCAGCA	loop
TTGGAACAAGAGTCCACTATTAAAGAACGTGGACTCCAACGT	loop
TTTGATGGTGGTTCCGAAATCGGCAAAATCCCTT	loop
ATAAATCAAAAGAATAGCCCGAGATAGGGTTGAGTGTTGTT	loop
AGCGGTCCACGCTGGTTTGCCCCAGCAGGCGAAAATCCTG	loop
CAAAGGGCGAAAAACCGTCTATCAGGGCGATGGCC	loop
TTTCTTTTCACCAGTGAGACGGGCAACAGCTGATT	loop
GAGAGGCGGTTTGCGTATTGGGCGCCAGGGTGGTT	loop
TCGTGCCAGCTGCATTAATGAATCGGCCAACGCGCGGG	loop
CACTACGTGAACCATCACCCAAATCAAGTTTTTTGGGG	loop
AATCATTCATACCGGGGGTACCAGTTTCCCCCAACAGGATTAGCAGAGCGAGG	protein attach YES
TTGAGTGGGTCAAAAGTAGAAGATTTCCCCCAACAGGATTAGCAGAGCGAGG	protein attach YES
CAGTGAGGCCACAAACGTTTCCCCCAACAGGATTAGCAGAGCGAGG	protein attach YES
ATCATCGTAGGAATCTTACACTACCGTTTCCCCCAACAGGATTAGCAGAGCGAGG	protein attach YES
TGAACACGATGCCGCGAATTTCCCCCAACAGGATTAGCAGAGCGAGG	protein attach YES
TCGCGTCCGTGAATCGTTTTCCCCCAACAGGATTAGCAGAGCGAGG	protein attach YES
GTGCTGCGGCCAGGCCATTTCCCCCAACAGGATTAGCAGAGCGAGG	protein attach YES
GGGGGTTTCATGGAGCTTATTAACAA	protein attach YES
GGGGGTTTGAAATAACGGCGCCAGTA	protein attach YES
GGGGGTTTCGAACCACCCTTCATAACCGCTACAGGGCGCGTA	protein attach YES
GGGGGTTTTCAAATGCGGTTGCGCAA	protein attach YES
GGGGGTTTAGAGACTGGTCAACAAAC	protein attach YES
GGGGGTTTACACGGTGGTGCTTAAATG	protein attach YES
GGGGGTTTGATAGCGCCTGCTCAGGAA	protein attach YES
GTTTTAGCGAACCTAACCGTCAGAATTTCCCCCAACAGGATTAGCAGAGCGAGG	protein attach YES
GGGGGTTTATATTTTGCAGGCCAACG	protein attach YES
GACAAAAGGTAACATGGTCTAACGGA	protein attach NO
TAATGCAGTTTTCGAAGCACCGTCGCT	protein attach NO
ATCGGCCAACAGTACTTTA	protein attach NO
CCGGCAGTTGTGTACATCGACATACTGATT	protein attach NO
CAAGAAAAGCCATATCGGCTGGCCGTT	protein attach NO
TAGAAACCAATCAAGTATAAAGCGCTTTTAGTG	protein attach NO
CGAACGATCCTGATTGTTTGGATTAAATCC	protein attach NO
GGCAGCGCAGAGACATCAAGAAAACATCTTCTGATAAGAG	protein attach NO
TCAAAAATCCGTGGGAGTTGGCAGGTTA	protein attach NO
ATTAAACTTCAGGCATGAGCCAACATT	protein attach NO
ATCATTATTCCTGATTATCAGATGA	protein attach NO
TCTAAATAACAGATGTGAGTGAATAAATATAACGGCGGAT	protein attach NO
TATTTTCCCGCTTCAAAACAGTTTGCC	protein attach NO
ACGTGCTTCGCCTTGAATTACCTTTTTAAGAACGTGAGCGAAGCCAGC	protein attach NO
ATGAAGGCCGCACAGGCGGCAGAAGGAGCGGTGAAGGG	protein attach NO
CAAACAGGGTTAGAACCTACCATATATTAG	protein attach NO
GTATTATACTTCACCTCACCGGAAACGCCAGGGT	protein attach NO
CAAAAGAACCCTCATATATTTTCGTCTTGCGTGCTTTACA	protein attach NO
TTTCTTTGCTCGGGTAAT	protein attach NO
AGAAGTCAAAATTAGATTAAGTTTCA	protein attach NO
TGCTTATACTTCTTTGAGCCTTG	protein attach NO
CGATGAAAAAATGGATCAAACTTAAAAAACACCGAGAATC	protein attach NO
GAAACCACCCTTCGCACTAATATCTGTAGCATCCTCGAGTCGGGAAACC	protein attach NO
CCTGCAGCCAGCCTATTA	protein attach NO
AAAGTTGGCAATACCAGTCCCGGAA	protein attach NO
AAAGAGTCTGTCCAGCCAT	protein attach NO
TTTTTTTTCAATTACCGTTGGGCGAACGCG	protein attach NO
GGAATGGGAGAAACAATAACGGACGGACTTGTAAA	protein attach NO
CACATTACCAAGTTACAAAATCGCCTCCGG	protein attach NO
GTTCAGCAAATCAACATC	protein attach NO
GTCTTTCTGAGAGCCAGCGGATTT	protein attach NO
GGGTACTGTTGCCCTGCATTTTA	protein attach NO
AGGGAACAGTTGAGAACGTCAGCACCAGT	protein attach NO
CTGGTCAATCCGCCGGGAACAAA	protein attach NO
AAAAGGGTCCAGCTGTGTTTTCTACAT	protein attach NO
GCCATTGGCGGTCAGTATCAACTC	protein attach NO
GCAAACGTAACCGAGGACCATATCCGTTTAATAAA	protein attach NO
TTGGCCAGCAACCGCAACCAGAG	protein attach NO
TGCAATCCAGCATCAGCGGTTAAA	protein attach NO
TTTGAGGTGCCATCCCACGTCCGT	protein attach NO
GGGGGTTTGAGAAGCCTAGTAATGTGTAGGTAAAGATTC	biotin anchors
GGGGGTTTAAGACTCCTTATTACGCATAGTAGCAT	biotin anchors
GGGGGTTTCATCGGCATACAGTCAAATCACCATCAATAT	biotin anchors
GGGGGTTTTGAAACCATCGATAGCAGCACCGTAATCAGTAGCGTAAATTAA	biotin anchors
GGGGGTTTGCAAAATCACCAGTAGCACCATTACCATTAGCAAGAGGCTAT	biotin anchors
GGGGGTTTTAAAGGTGAATTATCACCGTCACCGACTTGAGCCATCGATGA	biotin anchors
GGGGGTTTGACATTCAACCGATTGAGGGAGGGAAGGTAAATATGAGAGAT	biotin anchors
GATTAAGAGGAAGCCCGAAAGACTTCCGGAAGCAAGTCACCAATTTCCCCCTTT-TEG-Biotin	biotin anchors
AGGTCTTTACCCTGACTATTATAGTCGATAAGAGGTAGAGCCATTTCCCCCTTT-TEG-Biotin	biotin anchors
TAGCCCACATTATGACCCTGTAATACTTTTGCGGTTTCCCCCTTT-TEG-Biotin	biotin anchors
CTCAAATGCTTTAAACAGTTCAGAAAGTAGCTCAATTATTCATTTTCCCCCTTT-TEG-Biotin	biotin anchors
ACTGGATAGCGTCCAATACTGCGGAATCCATATAAAAAAGGGCTTTCCCCCTTT-TEG-Biotin	biotin anchors
TAGCAAAATCGCAAGGATAAAAATTTTTAGAACTGGCATGATTTTTCCCCCTTT-TEG-Biotin	biotin anchors
AGTTTGCCTTTAGCGTCAGACTGTAGCGCGTTTTTTTCCCCCTTT-TEG-Biotin	biotin anchors

Supplementary Table 3. Sequences of DNA origami ring with 32 protein attachment anchors and 7 biotin modifications.

	System	Median (G_{ring}/G_{pore})	95% Confidence interval [lower, upper bound]
Figure 4d	Ring	0.83	[0.8168, 0.8446]
	8-NSP1	0.77	[0.7581, 0.7731]
	32-Nsp1	0.66	[0.6503, 0.6750]
Figure 4e	Ring	0.88	[0.8724, 0.8779]
	8-NSP1-S	0.83	[0.8198, 0.8455]
	32-NSP1-S	0.79	[0.7821, 0.7984]
Figure 4f	Ring	0.86	[0.8574, 0.8706]
	8-NSP1-S	0.82	[0.8148, 0.8298]
	8-NSP1	0.80	[0.7800, 0.8139]
Figure 4g	Ring	0.85	[0.8348, 0.8567]
	32-NSP1-S	0.69	[0.6632, 0.7138]
	32-Nsp1	0.65	[0.6428, 0.6629]

Supplementary Table 4. Conductance values shown in Figure 4. Median relative conductance (G_{ring}/G_{pore}) and 95% confidence intervals for the bare DNA ring, the DNA ring coated with 8 and 32 copies of NSP1, and rings with NSP1-S.

System	G(d) nS	Stand. dev (G(d)) (nS)	Relative Conductance (RC)	Error (RC)
Bare ring	37.80	1.20	1.00	0.03
8-NSP1	33.53	1.01	0.89	0.04
8-NSP1-S	34.60	1.05	0.92	0.04
32-NSP1	26.15	0.79	0.69	0.04
32-NSP1-S	29.36	0.89	0.78	0.04

Supplementary Table 5. Computed conductance for DNA ring coated with 8 and 32 copies of NSP1 and NSP1-S.

System	G_{ring}/G_{pore}	Error	Relative Conductance (RC)	Error (RC)
Bare ring	0.85	0.03	1.00	0.04
8-NSP1	0.78	0.03	0.91	0.05
8-NSP1-S	0.83	0.02	0.97	0.04
32-NSP1	0.66	0.02	0.77	0.04
32-NSP1-S	0.74	0.07	0.87	0.10

Supplementary Table 6. Experimental conductance for DNA ring coated with 8 and 32 copies of NSP1 and NSP1-S.

Supplementary Note 1. Details of NSP1 and NSP1-S.

The NSP1 (67.5 kDa) and NSP1-S (62.1 kDa) were purified as described in Frey et al.[1,2].

Sequence of **NSP1**

MSKHHHHSGHHHTGHHHHSGSHHHTGENLYFQGSNFNTPQQNKTPFSFGTANNNSNTT
NQNSSTGAGAFGTGQSTFGFNNSAPNNTNNANSSITPAFGSNNTGNTAFGNSNPTSNVFG
SNNSTTNTFGSNSAGTSLFGSSSAQQTKSNGTAGGNTFGSSSLFNNSTNSNTTKPAFGGL
NFGGGNNTTPSSTGNANTSNNLFGATANANKPAFSFGATTNDDKKTEPDKPAFSFNSSVG
NKTDAQAPTTGFSFGSQLGGNKTVNEAAKPSLSFGSGSAGANPAGASQPEPTTNEPAKPA
LSFGTATSDNKTTNTTPSFSFGAKSDENKAGATSKPAFSFGAKPEEKKDDNSSKPAFSFGA
KSNEDKQDGTAKPAFSFGAKPAEKNNNETSKPAFSFGAKSDEKKDGDASKPAFSFGAKPD
ENKASATSKPAFSFGAKPEEKKDDNSSKPAFSFGAKSNEDKQDGTAKPAFSFGAKPAEKNN
NETSKPAFSFGAKSDEKKDGDASKPAFSFGAKSDEKKDSDSSKPAFSFGTKSNEKKDSGSS
KPAFSFGAKPDEKKNDEVSKPAFSFGAKANEKKESDESKSAFSFGSKPTGKEEGDGAKAAI
SFGAKPEEQKSSDTSKPAFTFGAQKDNEKKTETSC.

Sequence for **NSP1-S**

MSKHHHHSGHHHTGHHHHSGSHHHTGENLYFQGSNSNTPQQNKTPSSSGTANNNSNTT
NQNSSTGAGASGTGQSTSGSNNSAPNNTNNANSSSTPASGSNNTGNTASGNSNPTSNSS
GSNNSTTNTSGSNSAGTSSSGSSSAQQTKSNGTAGGNTSGSSSSSNNSTNSNTTKPASG
GSNSGGGNNTTPSSTGNANTSNNSSGATANANKPASSSGATTNDDKKTEPDKPASSSNSS
SGNKTDAQAPTTGSSSGSQSGGNKTSNEAAKPSSSSGSGSAGANPAGASQPEPTTNEPA
KPASSSGTATSDNKTTNTTPSSSSGAKSDENKAGATSKPASSSGAKPEEKKDDNSSKPASS
SGAKSNEDKQDGTAKPASSSGAKPAEKNNNETSKPASSSGAKSDEKKDGDASKPASSSGA
KPDENKASATSKPASSSGAKPEEKKDDNSSKPASSSGAKSNEDKQDGTAKPASSSGAKPA
EKNNNETSKPASSSGAKSDEKKDGDASKPASSSGAKSDEKKDSDSSKPASSSGTKSNEKK
DSGSSKPASSSGAKPDEKKNDESSKPASSSGAKANEKKESDESKSASSSGSKPTGKEEGD
GAKAASSSGAKPEEQKSSDTSKPASTSGAQKDNEKKTESTSC.

Supplementary Note 2. Calculation details of Supplementary Tables 5 and 6. Supplementary Table 5 shows the computed absolute conductance for the system with FG-nups tethered inside the DNA ring placed on a SiN nanopore (calculated using Eqn. 4) and bare DNA ring on a SiN nanopore (using Eqn. 1). The standard deviation in $G(d)$ accounts for the standard deviation in bare pore conductivity (σ_{bare}). We obtain the relative conductance and corresponding error by normalizing with the bare ring system. In order to compare the computational conductance with the experiments shown in Fig. 4d-g, we normalized the experimental relative conductance (averages of medians of different realizations from Fig. 4d-g) with the bare ring on a SiN nanopore results as shown in Supplementary Table 6.

Bibliography

1. Frey, S. & Görlich, D. A saturated FG-repeat hydrogel can reproduce the permeability properties of nuclear pore complexes. *Cell* **130,** 512–23 (2007).
2. Frey, S., Richter, R. P. & Görlich, D. FG-rich repeats of nuclear pore proteins form a three-dimensional meshwork with hydrogel-like properties. *Science* **314,** 815–7 (2006).
3. Kimanius, D., Forsberg, B., Scheres, S. & Lindahl, E. Accelerated cryo-EM structure determination with parallelisation using GPUs in RELION-2. *Elife* (2016).
